# A novel allosteric modulator of the cannabinoid CB_1_ receptor ameliorates hyperdopaminergia endophenotypes in rodent models

**DOI:** 10.1038/s41386-020-00876-5

**Published:** 2020-10-08

**Authors:** Catharine A. Mielnik, Kim S. Sugamori, David B. Finlay, Hayley H. A. Thorpe, Matthieu Schapira, Nirunthan Sivananthan, Chun Kit Li, Vincent M. Lam, Sean Harrington, Mostafa H. Abdelrahman, Laurent A. Trembleau, W. McIntyre Burnham, Jibran Y. Khokhar, Ali Salahpour, Amy J. Ramsey, Michelle Glass, Iain R. Greig, Ruth A. Ross

**Affiliations:** 1 University of Toronto, Faculty of Medicine, Department of Pharmacology & Toxicology, Toronto, ON Canada; 2 University of Otago, Department of Pharmacology & Toxicology, Dunedin, New Zealand; 3 University of Guelph, Department of Biomedical Sciences, Guelph, ON Canada; 4 Structural Genomics Consortium, University of Toronto, Toronto, ON Canada; 5 University of Aberdeen, Aberdeen, UK

**Keywords:** Pharmacology, Sensorimotor processing

## Abstract

The endocannabinoid system (eCBs) encompasses the endocannabinoids, their synthetic and degradative enzymes, and cannabinoid (CB) receptors. The eCBs mediates inhibition of neurotransmitter release and acts as a major homeostatic system. Many aspects of the eCBs are altered in a number of psychiatric disorders including schizophrenia, which is characterized by dysregulation of dopaminergic signaling. The GluN1-Knockdown (GluN1KD) and Dopamine Transporter Knockout (DATKO) mice are models of hyperdopaminergia, which display abnormal psychosis-related behaviors, including hyperlocomotion and changes in pre-pulse inhibition (PPI). Here, we investigate the ability of a novel CB_1_ receptor (CB_1_R) allosteric modulator, ABM300, to ameliorate these dysregulated behaviors. ABM300 was characterized in vitro (receptor binding, β-arrestin2 recruitment, ERK1/2 phosphorylation, cAMP inhibition) and in vivo (anxiety-like behaviors, cannabimimetic effects, novel environment exploratory behavior, pre-pulse inhibition, conditioned avoidance response) to assess the effects of the compound in dysregulated behaviors within the transgenic models. In vitro, ABM300 increased CB_1_R agonist binding but acted as an inhibitor of CB_1_R agonist induced signaling, including β-arrestin2 translocation, ERK phosphorylation and cAMP inhibition. In vivo, ABM300 did not elicit anxiogenic-like or cannabimimetic effects, but it decreased novelty-induced hyperactivity, exaggerated stereotypy, and vertical exploration in both transgenic models of hyperdopaminergia, as well as normalizing PPI in DATKO mice. The data demonstrate for the first time that a CB_1_R allosteric modulator ameliorates the behavioral deficits in two models of increased dopamine, warranting further investigation as a potential therapeutic target in psychiatry.

## Introduction

Dysregulation of dopaminergic and glutamatergic signaling are thought to underpin the development of psychosis and schizophrenia [1]. Pharmacological treatment of schizophrenia and psychosis includes the use of antipsychotics, which act as orthosteric receptor antagonists/partial agonists of various GPCR targets, including dopamine receptor D_2_ and serotonin receptor 5HT_1A_. However, antipsychotics are associated with extrapyramidal side effects, sedation, metabolic syndrome, and weight gain [2, 3].

The endocannabinoids, anandamide (AEA) and 2-arachidonoylglycerol (2-AG), are orthosteric agonists of the cannabinoid CB_1_ receptor (CB_1_R). CB_1_R are expressed presynaptically on various neuronal types, including GABAergic, glutamatergic and serotonergic neurons, where they mediate an inhibition of transmitter release. While not directly expressed on dopaminergic neurons, the endocannabinoid system acts as a crucial filter that integrates both inhibitory and excitatory signaling that modulates dopamine neuron signaling [4]. Furthermore, studies have shown that the endocannabinoid system is a negative modulator of both D_1_ and D_2_ receptor-mediated behaviors, implicating them in basal ganglia disorders [5]. As such, in combination with the complex dysregulation and circuit-based mechanisms for brain region-dependent alterations in dopaminergic signaling in psychiatry, this suggests that the CB_1_R may be a more attractive, alternative therapeutic target to the classical D_2_ receptor antagonism approaches of antipsychotics [4].

Furthermore, there is strong evidence from humans that both endocannabinoid levels and CB_1_R are dysregulated in schizophrenia [6–9]. Serum and CSF levels of AEA are higher in patients with schizophrenia at all stages of the illness and are normalized after treatment with antipsychotics. CB_1_R expression and binding is higher in post-mortem brain tissues of patients with schizophrenia [6, 9]. While the endocannabinoid system seems to be an important potential therapeutic target in psychiatry, targeting CB_1_R at the orthosteric site has not yielded beneficial clinical outcomes. The CB_1_R orthosteric inverse agonist, rimonabant, was effective treating obesity and metabolic syndrome, but caused suicidal ideation and was withdrawn from the market [10–12]. Here, we propose to investigate a novel pharmacological approach of targeting the CB_1_R.

In 2005, we discovered the CB_1_R allosteric site and the original, prototype allosteric modulator, Org27569. This compound has served as a tool compound to characterize the allosteric site but is not a drug candidate. Org275, and related compounds, display an atypical, complex allosteric profile at CB_1_R [13, 14]. Org275 *increases* the *B*_max_ of [^3^H] CB_1_R agonist binding but functionally acts as an *inhibitor* of CB_1_R agonist-mediated signaling [13, 15]. Importantly, in October 2019, Shao et al. [16] elucidated the ternary crystal structure of CB_1_R in complex with agonist and Org275. The structure shows that Org275 binds to a cholesterol-binding site on the CB_1_R, suggesting that the compound works by partitioning into the bilayer and competing with endogenous cholesterol for this surface. Previous studies have demonstrated that cholesterol may act as an endogenous modulator of CB_1_R [17]. There is growing evidence that, instead of targeting the orthosteric site of CB_1_R, the allosteric site may have key advantages [15, 18, 19]. By modulating the effects of the endogenous ligand, normal physiological tone (spatial and temporal effects of ligand binding to the receptor) are maintained, as opposed to the non-physiological binding and distribution seen with exogenous direct ligands such as orthosteric agonists or antagonists.

Since discovering the CB_1_R allosteric site in 2005, and identification of Org275 as the first CB_1_R-negative allosteric modulator [13], we, and others, have worked to develop both CB_1_R-negative and -positive allosteric modulators. The positive allosteric modulators developed by us, and others, have shown efficacy in the treatment of neuropathic pain [20] and other therapeutic indications [21]. As Org275, and related compounds, have insufficient metabolic stability, in order to further investigate the potential of this unique class of CB_1_R allosteric modulator, we embarked on a chemistry campaign, with the goal of generating new molecules with improved drug-like characteristics that are more suitable for in vivo testing and clinical development. Our working hypothesis is that the unique pharmacological profile of CB_1_R allosterics provides a distinctive pharmacological approach for modulation of the endocannabinoid system in complex disorders, and offers an alternative to CB_1_R orthosteric antagonists [22]. The in vivo outcomes of this complex mechanism are yet to be elucidated, particularly in models in which the endocannabinoid system is dysregulated.

Here, we present data on the effects of a novel CB_1_R allosteric modulator, ABM300, in two distinct transgenic mouse models, both of which present with a state of hyperdopaminergia. Both the GluN1-Knockdown (GluN1KD) and Dopamine Transporter Knockout (DATKO) mice have increased synaptic dopamine in subcortical regions [23–25], which is implicated in their phenotypic behavioral changes [24, 26–28], as well as disrupted sensorimotor gating [29–31].

## Methods and materials

### Animal ethics

Animal housing and experimentation were carried out in accordance with the Canadian Council in Animal Care (CCAC) guidelines for the care and use of animals and following protocols approved by the Faculty of Medicine and Pharmacy Animal Care Committee at the University of Toronto and the University of Guelph Animal Care Committee, respectively.

### Compound synthesis

See Supplementary Information for details.

### Pharmacokinetic analyses

Microsomal stability assays were conducted by Cyprotex Ltd (Macclesfield, UK). The in vitro metabolic stability of ABM300 was measured in the presence of human or rat liver microsomes by determination of the rate of compound disappearance. Single dose in vivo PK studies were conducted by Sai Life Ltd (Pune, India) to investigate the plasma pharmacokinetics and brain distribution of ABM300 in male C57Bl/6 mice following a single intraperitoneal (i.p.) administration of a 10 mg/kg dose.

### Predictive model of ABM300 bound to CB_1_R

The crystal structure of the CB_1_R-CP55940-Org275 complex (PDB code 6kqi) [16] was loaded into ICM (Molsoft, San Diego, CA), hydrogens were added, and rotameric states of hydroxy groups, histidine, asparagine, and glutamine side-chains optimized. ICM’s ligand editor was used to strip Org275 to the indole core scaffold shared with ABM300, and to incrementally grow the scaffold into ABM300, with a Monte Carlo-based energy minimization in the internal coordinate space at each step [32].

### Equilibrium-binding assays

Binding assays in hCB_1_R CHO cells were performed by Eurofins Cerep with the CB_1_R agonist, [^3^H]CP55,940 (0.5 nM, *K*_d_ of 3.5 nM). Non-specific binding was defined in the presence of 10 µM WIN55,212-2.

### PathHunter® β-arrestin assay

The PathHunter® β-Arrestin assay was conducted by Eurofins Pharma Discovery Services (further details can be found at https://www.eurofinsdiscoveryservices.com).

### ERK1/2 phosphorylation assay

CB_1_R-mediated ERK1/2 phosphorylation was quantified using an AlphaLISA® Surefire® Ultra™ pERK1/2 Assay (PerkinElmer, Woodbridge, ON) according to the manufacturer’s protocol, in hCB_1_R CHO cells plated at a density of 40,000 cells/well (96-well). ABM300 IC_50_ values were determined in the presence of increasing concentrations of ABM300 at the EC_80_ for CP55,940 (40 nM). Results are presented as the percent stimulation of ERK1/2 phosphorylation by CP55,940 alone.

### Cyclic AMP (cAMP) assays

A DiscoverX HitHunter® cAMP Assay for Small Molecules (DiscoverX, Fremont, CA) was used to quantify cAMP levels as per the manufacturer’s instructions. hCB_1_R CHO cells were seeded at a density of 40,000 cells/well (white 96-well), and after 24 h were serum-starved in the presence of 0.1% Bovine Serum Albumin for 60 min prior to pretreatment for 30 min with increasing concentrations of ABM300 (10^−10^–10^−5^ M) and a final 30 min incubation with 40 nM CP55,940 and 5 μM forskolin. The cAMP BRET experiments were performed as previously described [33, 34]. Briefly, hCB_1_R HEK293 cells were transfected with 5 µg/10 cm dish of the CAMYEL biosensor using polyethylenimine. After 24 h, the cells were plated into white 96-well plates (PerkinElmer) at a density of 60,000 cells/well.

### Drug administration

The following drugs were administered in a volume of 10 mL/kg via i.p. injections in a vehicle consisting of 95% ethanol, Tween80, and 0.9% NaCl in a 1:1:18 ratio, 30 min before behavioral testing, unless otherwise stated: ABM300 (10 mg/kg), rimonabant (RIM; 10 mg/kg, Cayman Chemical, Cat.# 9000484, Ann Arbor, MI), olanzapine (OLA; 1 mg/kg, Millipore Sigma, Cat.# O1141, Toronto, ON, CA), and Δ^9^-tetrahydrocannabinol (THC; 10 mg/kg, gift from MedReleaf, Markham, ON, CA).

### Cannabinoid-induced tetrad behaviors

Male C57Bl/6J mice (PD > 70) were tested on the cannabinoid-induced tetrad, as previously described [35].

### Behavioral testing in murine models of hyperdopaminergia

The effect of ABM300 was compared to OLA. GluN1KD (F1 on C57Bl/6J x 129/SvlmJ background) [26] and DATKO (C57Bl/6J background) [24] mice were used as murine models of hyperdopaminergia. All mice were tested as follows: Day 1—open-field test and Day 3—pre-pulse inhibition, as previously described [28, 30, 36, 37].

### Quantification and statistical analysis

For in vitro assays, results were analyzed by non-linear regression analysis of sigmoidal dose-response curves. For in vivo assays, statistical parameters, the definition of measures and statistical significance are reported in the figures and the figure legends. Data are represented as mean ± SEM. Studies and data analysis were not blinded. Differences in means were considered statistically significant at *p* < 0.05. All data analyses were performed using GraphPad Prism 6.0 or 8.0 software (San Diego, CA) and/or IBM SPSS 23.0 Software (Armonk, NY).

## Results

### Pharmacokinetics of ABM300

ABM300 (5-(5-chloro-3-ethyl-1H-indol-2-yl)-*N*-phenyl-1,3,4-oxadiazol-2-amine) showed promising in vitro metabolic stability in human and rat liver microsomal preparations with half-life values of 109 and 110 min (compared to a typical developmental target for progression of  > 45 to 60 min), respectively (CL_int_ = 12.7 ± 3.4 and 12.6 ± 2.0 µL/min/mg protein). Subsequent in vivo pharmacokinetic studies in male C57Bl/6 mice (8 timepoints, *N* = 3 per timepoint) confirmed acceptable metabolic stability for our studies (*T*_1/2_ ~2 h) and CNS exposure, with a brain to plasma ratio of 0.77 from a dose of  10 mg/kg, i.p., (AUC_8h brain_/AUC_8h plasma_) and brain concentrations of 374 ± 39 ng/mL at 30 min (Supplementary Fig. S1 and Supplementary Table S1).

### Docking model of ABM300 bound to CB_1_R

**Fig. 1:**
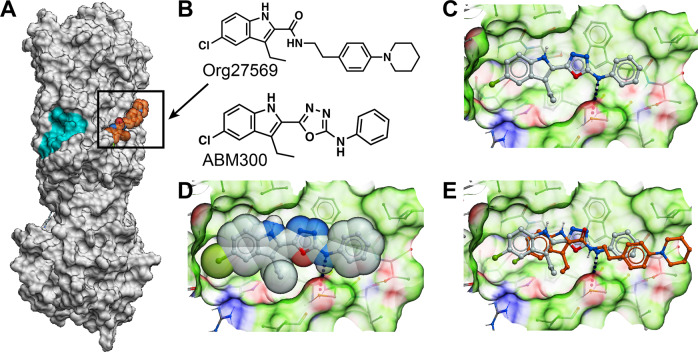
Molecular structure and docking model of ABM300 bound to CB_1_R. **a** Overall structure of CB_1_R (PDB code: 6kqi) with bound Org275 (orange). The pregnenolone allosteric-binding site is highlighted in cyan [60], **b** Molecular structures of ABM300 and Org275. **c** Overall model showing ABM300 and surrounding side-chains. **d** Space filling representation showing that ABM300 occupies optimally the negative allosteric modulator-binding pocket. **e** ABM300 superimposed with the crystal structure of Org275 (orange) bound to CB_1_R (PDB code: 6kqi). The molecular surface of CB_1_R is color-coded based on binding properties. Green: hydrophobic. Red: hydrogen-bond acceptor. Blue: hydrogen-bond donor.

A model of ABM300 bound to CB_1_R was derived from the CB_1_-CP55940-Org275 ternary complex [16] (Fig. 1). ABM300 recapitulates hydrophobic interactions observed with Org275 in the crystal structure. Owing to its increased rigidity, ABM300 needs to shift by 2 Å towards the wall formed by I141 to accommodate the phenyl ring that abuts I245. The docked conformation optimally occupies the pocket, the oxadiazole ring is stacked against the indole of W241, and the secondary amine bridging the phenyl and oxadiazole rings is engaged in a hydrogen-bond with C238.

### In vitro pharmacology: ABM300 increases agonist binding but inhibits CB_1_R orthosteric agonist signaling

**Fig. 2:**
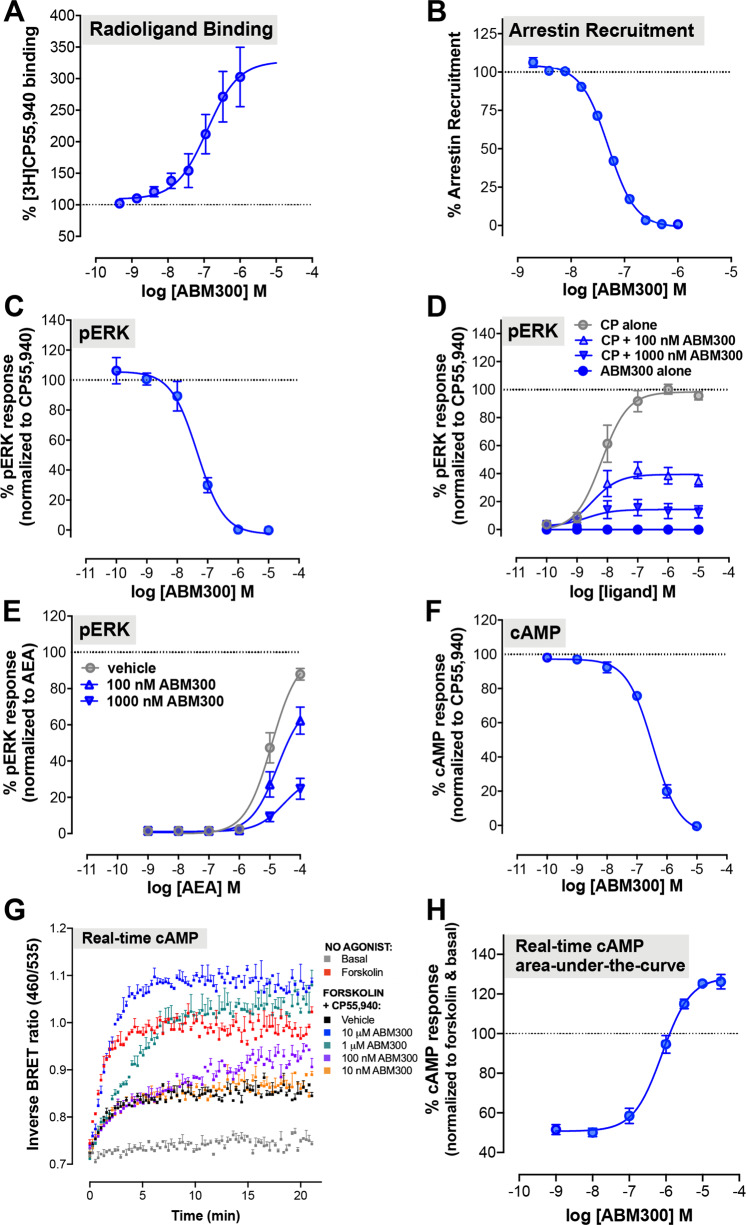
ABM300 increases agonist binding but inhibits CB_1_R orthosteric agonist signaling through arrestin recruitment, ERK phosphorylation and cAMP signaling. **a** ABM300 increases [^3^H]CP55,940 binding to hCB_1_R CHO cell membranes. **b** ABM300 concentration-dependently decreases CP55,940 (10 nM)-mediated arrestin recruitment, with the PathHunter® β-arrestin assay. **c** ABM300 concentration-dependently decreases ERK phosphorylation at the EC_80_ concentration of CP55,940 (40 nM), using the AlphaScreen® SureFire® ERK1/2 phosphorylation kit in hCB_1_R CHO cells. **d** ABM300 has no effect alone, but decreases the *E*_max_ for CP55,940-stimulated ERK phosphorylation in a concentration-dependent manner in hCB_1_R CHO cells. **e** ABM300 decreases the *E*_max_ of AEA-stimulated ERK1/2 phosphorylation in a concentration-dependent manner in hCB_1_R CHO cells. **f** ABM300 concentration-dependently inhibits CP55,940-(EC_80_ of 40 nM) mediated inhibition of forskolin-stimulated cAMP signaling in hCB_1_R CHO cells. Data shown as mean ± SEM from 3–5 independent experiments conducted in triplicate. **g** BRET CAMYEL real-time cAMP signaling data in hCB_1_R HEK cells, showing ABM300 concentration-dependently inhibiting the reduction in cAMP level induced by 5 µM forskolin and 1 µM CP55,940; the time-dependent activity of ABM300 is particularly apparent at moderate concentrations (1 µM and 100 nM), in which the onset of the ABM300 effect is delayed (representative experiment). **h** Area-under-the-curve analysis of **g**, showing that ABM300 concentration-dependently inhibits CP55,940-mediated cAMP reductions in HEK cells. At high concentrations of ABM300, cAMP levels are increased above forskolin alone (100%).

In line with our previous studies using a related compound (Org275) [13], we find that ABM300 causes a significant and concentration-dependent increase in the specific binding of [^3^H]CP55,940 to hCB_1_R CHO (Fig. 2a) with an *E*_max_ value of 328 ± 47% and a EC_50_ value of 132 nM (pEC_50_ 6.90 ± 0.09) and an α value of 4.33 ± 0.79 (logα 0.622 ± 0.08) (Supplementary Table S2).

In the PathHunter® β-arrestin CB_1_R assay, CP55,940-stimulated β-arrestin recruitment with an EC_50_ value of 5.37 nM (pIC_50_ 8.28 ± 0.06) and an *E*_max_ of 104.3 ± 0.90%. In the presence of the EC_80_ of CP55,940 (10 nM), ABM300 produced a concentration-related reduction in β-arrestin recruitment with an IC_50_ value of 49.7 nM (pIC_50_ 7.31 ± 0.02) (Fig. 2b).

Using an AlphaScreen® SureFire® ERK1/2 phosphorylation assay kit, we measured the effect of ABM300 on activation of ERK1/2 phosphorylation by CP55,940 in hCB_1_R CHO cells. In the presence of vehicle, CP55,940 induced ERK1/2 phosphorylation with an EC_50_ of 12.1 nM (pEC_50_ 8.14 ± 0.23) (Fig. 2c). At concentrations of 100 or 1000 nM, ABM300 significantly decreased CP55,940 *E*_max_ (efficacy), to 40.3 ± 5.57% and 14.7 ± 5.22%, respectively (Fig. 2c). ABM300 alone did not affect ERK1/2 phosphorylation at concentrations up to 10 µM (Fig. 2c). Additionally, 100 or 1000 nM ABM300 significantly decreased the *E*_max_ (efficacy) of AEA from 100.3 ± 0.20% to 80.23 ± 14.4% and 31.88 ± 6.09%, respectively (Fig. 2d). In the presence of the EC_80_ (40 nM) of CP55,940, ABM300 produced a concentration-related reduction in ERK1/2 phosphorylation with an IC_50_ value of 47.0 nM (pIC_50_ 7.38 ± 0.12) (Fig. 2e). CP55,940 inhibited forskolin-stimulated cAMP accumulation in hCB_1_R CHO cells (data not shown). At CP55,940 EC_80_ (40 nM), ABM300 blocked this inhibition with an IC_50_ value of 379 nM (pIC_50_ 6.45 ± 0.08) (Fig. 2f).

We further characterized the real-time kinetic effect of ABM300 using a cAMP BRET sensor assay in HEK293 cells. Similar to previous observations with Org275 [38], ABM300 produced a complex, concentration and time-dependent modulation of agonist-mediated regulation of cAMP levels (Fig. 2g, h). Levels of cAMP were measured over time with a high concentration of CP55,940 (1 μM) in the presence of varying concentrations of ABM300. Consistent with Org275 observations, ABM300 did not affect the initial inhibition of cAMP by CP55,940 but, following a concentration-dependent “lag” in drug onset, inhibition of the agonist effect became apparent. At high concentrations, the ABM300 inhibitory effect overcame the CP55,940 effect and further enhanced cAMP levels above those produced by forskolin alone—reflecting inverse agonism.

### ABM300 does not bind to the CB_2_R and does not have off-target effects in a Safety Screen® 44 panel

Off-target effects of ABM300 were assessed using the SafetyScreen44, conducted at Eurofins Discovery Services. The screen assesses the selectivity of the compound on a diverse panel of targets that includes GPCRs, drug transporters, ion channels, nuclear receptors, kinases, and other non-kinase enzymes. The screen employs radioligand binding or enzyme assays for these 44 targets. At 1 µM, ABM300 did not display any significant binding to these targets, including; receptors CB_2_, Dopamine D_1_ and D_2_, NMDA, 5HT_1A_, 5HT_1B_, 5HT_2A_, 5HT_2B_, and 5HT_3_ (Supplementary Fig. S2).

### ABM300 has no effect in the cannabinoid-induced tetrad alone and does not display anxiogenic-like effects

**Fig. 3:**
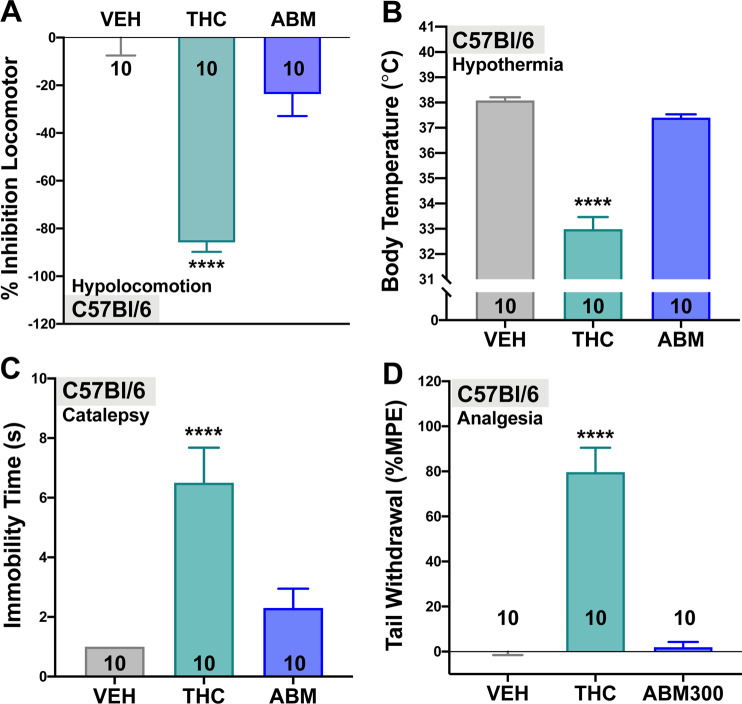
ABM300 (10 mg/kg) has no effect in the cannabinoid-induced tetrad, when compared to THC (10 mg/kg). Cannabinoid-induced tetrad measuring **a** percent inhibition of locomotor activity (15 min), **b** rectal temperature (°C), **c** catalepsy-induced immobility time (s), and **d** tail withdrawal (% MPE). ABM300 (10 mg/kg) alone has no effect on all outputs, when compared to vehicle and THC. All tests performed in male mice, ABM300 or THC were administered 30 min prior to behavioral testing via i.p. injection. Data shown as mean ± SEM, *p ≤ 0.05 compared to vehicle, ****p < 0.0001, one-way ANOVA, multiple comparisons, post-hoc Sidak’s test. Effect of treatment **a** *F*[2,27] = 37.47, *p* < 0.0001, **b** *F*[2,27] = 85.58, *p* < 0.0001, **c** *F*[2,27] = 13.72, *p* < 0.0001, and **d** *F*[2,26] = 46.67, *p* < 0.0001.

We confirmed that ABM300 (10 mg/kg) did not have agonist activity via the cannabinoid-induced tetrad, compared to THC (10 mg/kg). In all four tetrad measures (Fig. 3), THC, but not ABM300, produced effects (*p* < 0.0001 for all outputs). Previous reports demonstrated adverse effects observed with CB_1_R orthosteric agonists/inverse agonists [39, 40]. Possible anxiogenic effects of ABM300 (10 mg/kg) were investigated using the EPM and compared to rimonabant (10 mg/kg) (Supplementary Fig. S3). ABM300 did not affect time spent in the open arms (*p* = 0.3335), whereas rimonabant significantly reduced open arm time compared to vehicle (*p* = 0.0059).

### ABM300 decreases novelty-induced hyperactivity, exaggerated stereotypy, and vertical exploration in GluN1KD mice

**Fig. 4:**
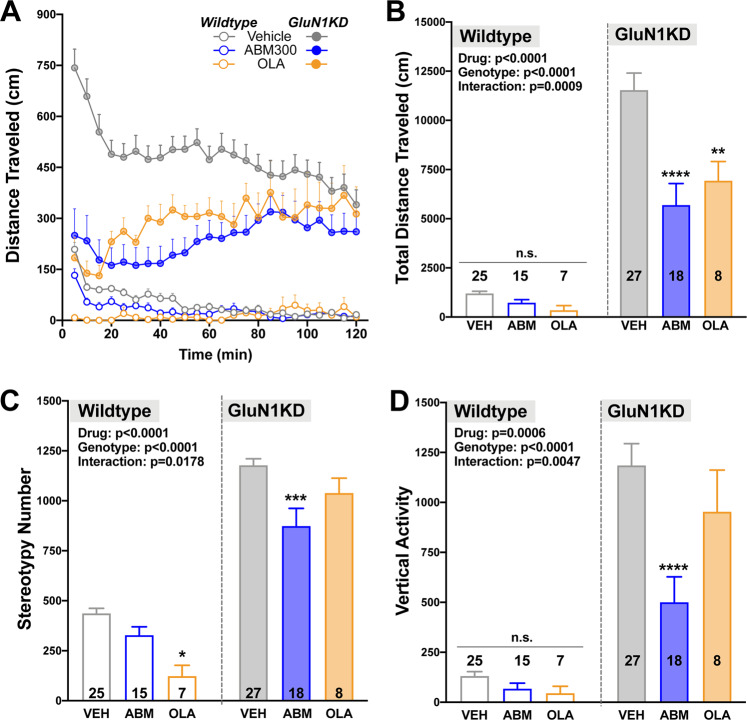
ABM300 corrects hyperactivity, aberrant stereotypic movements and rearing behavior, resulting from hyperdopaminergia in the GluN1KD mouse model. ABM300 (ABM; 10 mg/kg) decreases novelty-induced hyperactivity (time-course of distance traveled—**a**, total distance traveled—**b**) aberrant stereotypic movements (**c**), and mania-like rearing behavior (**d**), in the open-field test. Effects of ABM300 are similar to those seen with olanzapine (OLA—1 mg/kg). All tests balanced for sex, drugs administered 30 min before test via i.p. injection. Data shown as mean ± SEM, *p ≤ 0.05 compared to vehicle (within genotype), *p < 0.05, **p < 0.01, ***p < 0.001, ****p < 0.0001, two-way ANOVA, multiple comparisons, post-hoc Sidak’s test. **a**, **b** Effect of genotype *F*[1,94] = 100.7, *p* < 0.0001, effect of drug *F*[2,94] = 11.23, *p* < 0.0001, interaction of genotype x drug *F*[2,94] = 7.554, *p* = 0.0009. **c** Effect of genotype *F*[1,94] = 223.6, *p* < 0.0001, effect of drug *F*[2,94] = 12.13, *p* < 0.0001, interaction of genotype x drug *F*[2,94] = 4.206, *p* = 0.0178. **d** Effect of genotype *F*[1,94] = 70.87, *p* < 0.0001, effect of drug *F*[2,94] = 8.137, *p* = 0.0006, interaction of genotype x drug *F*[2,94] = 5.679, *p* = 0.0047.

GluN1KD mice display hyperactivity, increased stereotypy and vertical exploration patterns, along with impairment in sensorimotor gating [26–29]. GluN1KD mice do not display a difference in *Cnr1* mRNA expression in key brain regions mediating these behaviors (Supplementary Fig. S4). ABM300 decreased the number of dysregulated behaviors in the GluN1KD model of hyperdopaminergia (Fig. 4). Hyperactivity (Fig. 4a, b) was affected by genotype (*F*[1,94] = 100.7, *p* < 0.0001), and GluN1KD mice responded to ABM300 (*p* < 0.0001) when compared to vehicle. Significant effects of genotype were observed for stereotypy and vertical exploration (Fig. 4c, d) (*F*[1,94] = 223.6, *p* < 0.0001; *F*[1,94] = 70.87, *p* < 0.0001, respectively). ABM300 significantly reduced exaggerated stereotypic activity (Fig. 4c), as well as phenotypic increased rearing behavior (Fig. 4d) (*p* < 0.0001). Sensorimotor gating deficits, along with acoustic startle response, did not respond to ABM300 or the atypical antipsychotic olanzapine (*4* *dB*: *F*[2,95] = 1.068, *p* = 0.3476; *8* *dB*: *F*[2,95] = 0.4576, *p* = 0.6342; *16* *dB*: *F*[2,95] = 0.4790, *p* = 0.6209; *ASR:* *F*[2,95] = 3.016, *p* = 0.0537) (Supplementary Fig. S5).

### ABM300 decreases novelty-induced hyperactivity, exaggerated stereotypy, vertical exploration, and normalizes pre-pulse inhibition (PPI) in DATKO mice

**Fig. 5:**
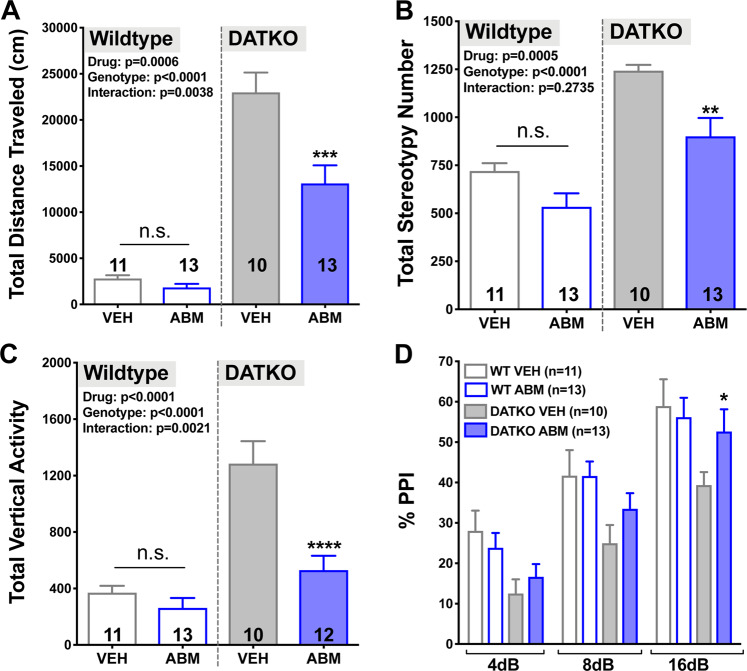
The effectiveness of ABM300 is recapitulated in a second, distinct, mouse model of hyperdopaminergia, the DATKO model, with additional restoration of sensorimotor deficits. ABM300 (10 mg/kg) decreases novelty-induced hyperactivity (total distance traveled; cm) (**a**), aberrant stereotypic movements (**b**), and mania-like vertical exploration (**c**) in the open-field test. ABM300 ameliorates sensorimotor gating deficits (**d**), rescuing the PPI deficit at 16 dB pre-pulse. All tests balanced for sex, drugs administered 30 min before test via i.p. Data shown as mean ± SEM, *p ≤ 0.05 compared to vehicle (within genotype), *p < 0.05, **p < 0.01, ***p < 0.001, ****p < 0.0001, two-way ANOVA, multiple comparisons, post-hoc Sidak’s test. **a** Effect of genotype *F*[1,43] = 116.9, *p* < 0.0001, effect of drug *F*[1,43] = 13.89, *p* = 0.0006, interaction of genotype x drug *F*[1,43] = 9.384, *p* = 0.0038. **b** Effect of genotype *F*[1,43] = 40.18, *p* < 0.0001 and effect of drug *F*[1,43] = 14.22, *p* = 0.0005, **c** Effect of genotype *F*[1,42] = 36.03, *p* < 0.0001, effect of drug *F*[1,42] = 19.16, *p* < 0.0001, interaction of genotype x drug *F*[1,42] = 10.80, *p* = 0.0021, **d** (4 dB) effect of genotype *F*[1,43] = 8.509, *p* = 0.0056, (8 dB) effect of genotype *F*[1,43] = 7.303, *p* = 0.0098, (16 dB) effect of genotype *F*[1,43] = 4.713, *p* = 0.0355.

The DATKO mouse, another model of hyperdopaminergia, displays hyperactivity, increased stereotypy and vertical exploration, with an impairment in sensorimotor gating [24, 31]. Similar to the GluN1KD model, DATKO mice showed no change in *Cnr1* mRNA expression (Supplementary Fig. S6). ABM300 normalized dysregulated hyperactivity, stereotypic movements, vertical exploration and sensorimotor gating in the DATKO mice (Fig. 5), a pattern of findings similar to what was seen in the GluN1KD model (Fig. 4). For the hyperactivity measure (Fig. 5a), a significant interaction was found between ABM300 and genotype (*F*[1,46] = 9.38, *p* = 0.004), indicating that ABM300 has genotype-specific effects on the exacerbated hyperactivity endophenotype in DATKO. There was an effect of genotype on exaggerated stereotypic movements and mania-like rearing behavior (*F*[1,46] = 40.18, *p* < 0.001; *F*[1,46] = 26.50, *p* < 0.001, respectively), which ABM300 had a beneficial effect in decreasing (*F*[1,46] = 14.22, *p* < 0.001; *F*[1,46] = 7.38, *p* = 0.009, respectively) (Fig. 5b, c). Furthermore, the actions of ABM300 extended to sensorimotor gating deficits present in DATKO (Fig. 5d), with a rescue of the PPI deficit at the 16 dB pre-pulse interval (*p* = 0.018). Neither genotype, nor treatment with ABM300, had an effect on the acoustic startle response in the DATKO model (data not shown, genotype: *p* = 0.214, ABM300: *p* = 0.516).

### ABM300 has no effect on the conditioned avoidance response (CAR)

The CAR task has been used as a test to infer antipsychotic efficacy via the selective suppression of the avoidance response [41, 42]. Administration of olanzapine (1 mg/kg), but not ABM300 (10 mg/kg), attenuated avoidance behavior in CAR testing (Supplementary Fig. S7). A main effect of drug was observed (*p* = 0.007). Furthermore, olanzapine, but not ABM300, enhanced escape responding during CAR (Supplementary Fig. S7B), with a main effect of drug treatment (*p* = 0.011) and an interaction between drug treatment and testing day (*p* = 0.034). Lastly, neither ABM300 nor olanzapine affected escape failures (Supplementary Fig. S7C). ABM300 did not induce catalepsy, or changes in body temperature (Supplementary Fig. S7D,E).

## Discussion

There is growing interest in the possible therapeutic potential of CB_1_R allosteric molecules [21]. Here, we show for the first time that a novel CB_1_R allosteric modulator ameliorates select disrupted behaviors in two distinct models of hyperdopaminergia. The rescue of these phenotypes by ABM300 occurred without adverse anxiogenic-like or cannabimimetic effects traditionally observed with orthosteric CB_1_R inverse agonists. Thus, these findings represent the first study demonstrating the potential for the use of a CB_1_R allosteric modulator as a therapeutic strategy for the treatment of hyperdopaminergic states, such as psychosis and mania.

Our molecular docking data indicate that ABM300 binds to the recently elucidated binding site for the original allosteric modulator Org275 [16]. The Org275-binding site on CB_1_R overlaps with a cholesterol-binding site, which is an extrahelical site within the inner leaflet of the membrane. The model is broadly in agreement with literature demonstrating that Org275 apparently stabilizes a high affinity, agonist bound CB_1_R [43], which may impede activation of selected downstream signaling pathways. The structure proposes a mechanism by which Org275, binding to the cholesterol site, captures an intermediate conformation that binds the orthosteric agonist and also inhibits G protein coupling. The model accommodates the complex effects of Org275 on CB_1_ orthosteric ligand binding, including the increase in *B*_max_ of agonists. ABM300 displays a similar in vitro pharmacological profile to Org275 [13, 15], whereby it increases CB_1_R agonist binding with an α value > 1 (4.33 ± 0.79) [44], but acts as a functional inhibitor of CB_1_R agonist-mediated β-arrestin recruitment (IC_50_, 50 nM), ERK phosphorylation (IC_50_, 47 nM), and cAMP inhibition (IC_50_, 380 nM).

As previously described [38], the unique time-dependent mechanism of action of ABM300 (Fig. 2g) posits that at moderate concentrations (high nM/low µM), early agonist signaling may remain unaffected; with inhibition initiating after a lag. These observations indicate that the molecular mechanism of action of ABM300 is related to previously characterized CB_1_R allosteric modulators such as Org275 and PSNCBAM-1 [38]. This may introduce potential for a unique kinetic profile of modulation of endocannabinoid signaling. The consequences of such effects at a network level and in a disease state are highly complex but may underlie the beneficial effects observed with ABM300 in the genetic models of hyperdopaminergia. Furthermore, expression levels, affinity, and pre-coupling of CB_1_Rs can significantly differ in various neuronal cell types; CB_1_R on GABAergic interneurons have significantly higher agonist affinity than those found on glutamatergic terminals, but the coupling efficacy of glutamatergic CB_1_R is significantly higher [45, 46]. This expression and affinity profile leads to the complex nature in the function of the endocannabinoid system, which explains the diverse effects of certain cannabinoid drugs, and the opposing effects in different illnesses [46]. We hypothesize that, in the genetic murine models of hyperdopaminergia, ABM300 acts as a modulator of endogenous CB_1_R signaling in vivo and, potentially, selectively modulates the endocannabinoid system in specific neurotransmitter system pathways to ameliorate the dysregulated behaviors observed in these mice.

Studies have demonstrated that administration of compounds that increase endocannabinoid levels modulate a number of schizophrenia-like responses in mice [47]. Studies in cultured cells have shown that the related compound, Org275, causes migration of CB_1_R to the soma, while cholesterol, which binds to the same site as ABM300 and acts as a positive modulator, allows for the enrichment of CB_1_R at the axon [48]. Thus, in addition to complex effects on endocannabinoid affinity and signaling, there is the potential for the modulation of topological CB_1_R membrane localization by CB_1_R allosterics. The consequences of this in a complex neuronal network, and pathological states, are yet to be fully elucidated.

To assess psychosis-like behaviors, and therapeutic efficacy of ABM300, we focused on two main behavioral categories: exploratory behavior (hyperactivity) and sensorimotor gating (disruption in PPI). The literature related to psychosis-like behaviors in genetically modified mouse models has classically focused on the same two categories [49]. Although these behaviors do not directly translate to human symptoms present in the disease, they do mimic the neurotransmitter changes that are involved in psychotic symptoms. It has been accepted that subcortical hyperdopaminergia is implicated in psychosis, confirmed further by the neuropharmacological action of antipsychotic drugs currently available; all licensed pharmacological treatments of psychosis (antipsychotics) require interactions with the dopamine D_2_ receptor [50]. Therefore, by assessing locomotor behavior within this study, we can infer that, driven by subcortical dopamine levels, an increase in dopamine leads to enhanced motor activity (either horizonal, rearing, and/or stereotypy) [49]. Meanwhile, disruption in PPI allows for a more straightforward phenotypic analysis of psychosis, as it has been reported in a number of psychiatric diseases, particularly schizophrenia and psychosis [51].

Here, we show that ABM300 restores dysregulated dopamine-mediated exploratory activity in both genetic models: decreasing exaggerated hyperactivity, stereotypy and rearing. Furthermore, ABM300 rescues PPI deficits in the DATKO model. Since psychosis symptomology is never present alone in a disease state such as schizophrenia, it would be intriguing to investigate the effects of ABM300 in other symptomatic domains, such as cognition. CB_1_R antagonism and loss of function may enhance some forms of learning and memory, and it is possible that CB_1_R-negative allosteric modulators may be pro-cognitive in preclinical schizophrenic models [52, 53].

We saw no effect of the compound in CAR in rat even though olanzapine produced significant suppression of CAR as has been shown in previous studies [54]. CAR is an extensively validated preclinical test used to predict therapeutic efficacy of antipsychotics that directly target the dopaminergic and serotoninergic receptor systems [42, 55]. This experiment further supports our assertion that ABM300’s antipsychotic effects are not mediated via dopamine D2 receptors, as considerable occupancy of striatal D2 receptors is required to see suppression of CAR (65–80% for typical antipsychotics, ~ 50% for atypical antipsychotics like clozapine) [42].

It is important to note that we do not have direct evidence for the involvement of the CB_1_R in the in vivo effect of ABM300 in genetic models. Directly implicating CB_1_R is challenging. One approach would be to create a double knockout of CB_1_R-/- and DAT-/- or GluN1-/-. However, given the crucial role of the CB_1_R in dopaminergic and glutamatergic signaling, there is a strong likelihood that the double knockouts could have a novel phenotype (such as seizures) or that previous phenotypes would be exacerbated [56, 57]. Furthermore, genetic manipulation of the CB_1_R has been shown to have effects on dopamine signaling [58, 59]. Another approach would be to investigate whether a CB_1_R orthosteric antagonist would block the effects of ABM300, thereby implicating CB_1_R. However, there is also doubt as to whether an orthosteric CB_1_R antagonist would block the effects of a negative allosteric inhibitor (antagonist blocking inhibitor); it is conceivable that a synergistic effect might be observed. Taken together, these limitations highlight the complexity of directly implicating CB_1_R in the mechanism of action of ABM300 in vivo in these genetic models. We plan to make this the subject of future investigations involving a variety of in vivo and ex vivo approaches. The pharmacological profiling and assessment of the potential for off-target interactions of ABM300 in binding screens suggest that the compound is CB_1_R-selective. Taken together, the potent inhibitory effect of the compound on CB_1_R signaling at nM concentrations in vitro and the favorable PK and brain penetration data, there is substantial support for the hypothesis that the CB_1_R mediates the effects of ABM300 in vivo in the genetic mouse models of hyperdopaminergia. It is also notable that the present study only included acute administration of one dose of ABM300. Future experiments will focus on various dose regimens, chronic dosing, development of tolerance and the effects of ABM300 in combination with an antipsychotic.

The data presented here offer the first evidence that acute administration of a CB_1_R allosteric modulator, with a unique pharmacological profile, effectively ameliorates certain behavioral deficits in two distinct models of increased dopamine. These data highlight that the allosteric-binding pocket on the CB_1_R warrants further investigation as a potentially important therapeutic target in psychiatry.

## Funding and disclosure

The authors declare the following financial and biomedical conflict of interests: Ruth A. Ross, Catharine A. Mielnik, Amy J. Ramsey, Iain R. Greig, Laurent A. Trembleau, Mostafa H. Abdelrahman are co-inventors on a patent application related to ABM300 and structural analogs. Kim S. Sugamori, David B. Finlay, Hayley H.A. Thorpe, Matthieu Schapira, Nirunthan Sivananthan, Chun Kit Li, Vincent M. Lam, Sean Harrington, W. McIntyre Burnham, Jibran Y. Khokhar, Ali Salahpour, Michelle Glass reported no biomedical financial interests or potential conflicts of interest. W. McIntyre Burnham received Δ9- (THC) as a gift from MedReleaf. The authors would like to gratefully acknowledge Wendy Horsfall for mouse colony maintenance. The work was funded by grants to RAR from CIHR (PPP-125784, PP2-139101), CIHR funding to AJR (MOP119298) and CIHR funding to AS (PJT-15619).

## Supplementary information

Mielniketal2020_Supplemental Material

## References

[1] Lyne J, Kelly BD, O’Connor WT (2004). Schizophrenia: a review of neuropharmacology. J Med Sci..

[2] Newcomer JW, Haupt D (2006). The metabolic effects of antipsychotic medications. Can J Psychiatry.

[3] Xu H, Zhuang X (2019). Atypical antipsychotics-induced metabolic syndrome and nonalcoholic fatty liver disease: a critical review. Neuropsychiatr Dis Treat.

[4] Covey DP, Mateo Y, Sulzer D, Cheer JF, Lovinger DM (2017). Endocannabinoid modulation of dopamine neurotransmission. Neuropharmacology..

[5] Martín AB, Fernandez-Espejo E, Ferrer B, Gorriti MA, Bilbao A, Navarro M (2008). Expression and function of CB1 receptor in the rat striatum: localization and effects on D1 and D2 dopamine receptor-mediated motor behaviors. Neuropsychopharmacology..

[6] Volk DW, Lewis DA (2016). The role of endocannabinoid signaling in cortical inhibitory neuron dysfunction in schizophrenia. Biol Psychiatry.

[7] Leweke FM, Mueller JK, Lange B, Fritze S, Topor CE, Koethe D (2018). Role of the endocannabinoid system in the pathophysiology of schizophrenia: implications for pharmacological intervention. CNS Drugs.

[8] Jacobson MR, Watts JJ, Boileau I, Tong J, Mizrahi R. A systematic review of phytocannabinoid exposure on the endocannabinoid system: Implications for psychosis. Eur Neuropsychopharmacol. 2019 10.1016/j.euroneuro.2018.12.014. 10.1016/j.euroneuro.2018.12.014 30635160

[9] Minichino A, Senior M, Brondino N, Zhang SH, Godwlewska BR, Burnet PWJ (2019). Measuring disturbance of the endocannabinoid system in psychosis: a systematic review and meta-analysis. JAMA Psychiatry..

[10] Kelly DL, Gorelick DA, Conley RR, Boggs DL, Linthicum J, Liu F (2011). Effects of the cannabinoid-1 receptor antagonist rimonabant on psychiatric symptoms in overweight people with schizophrenia: A randomized, double-blind, pilot study. J Clin Psychopharmacol.

[11] Christensen R, Kristensen PK, Bartels EM, Bliddal H, Astrup A (2007). Efficacy and safety of the weight-loss drug rimonabant: a meta-analysis of randomised trials. Lancet..

[12] Moreira FA, Grieb M, Lutz B (2009). Central side-effects of therapies based on CB1 cannabinoid receptor agonists and antagonists: focus on anxiety and depression. Best Pract Res Clin Endocrinol Metab..

[13] Price MR, Baillie GL, Thomas AAA, Stevenson LA, Easson M, Goodwin R (2005). Allosteric modulation of the Cannabinoid CB1 receptor. Mol Pharm.

[14] Baillie GL, Horswill JG, Anavi-Goffer S, Reggio PH, Bolognini D, Abood ME (2013). CB1 receptor allosteric modulators display both agonist and signaling pathway specificity. Mol Pharm.

[15] Ross RA (2007). Allosterism and cannabinoid CB1 receptors: the shape of things to come. Trends Pharm Sci.

[16] Shao Z, Yan W, Chapman K, Ramesh K, Ferrell AJ, Yin J, et al. Structure of an allosteric modulator bound to the CB1 cannabinoid receptor. Nat Chem Biol. 2019 10.1038/s41589-019-0387-2. 10.1038/s41589-019-0387-2 31659318

[17] Bari M, Battista N, Fezza F, Finazzi-Agrò A, Maccarrone M (2005). Lipid rafts control signaling of type-1 cannabinoid receptors in neuronal cells: Implications for anandamide-induced apoptosis. J Biol Chem.

[18] Nguyen T, Li JX, Thomas BF, Wiley JL, Kenakin TP, Zhang Y (2017). Allosteric modulation: an alternate approach targeting the cannabinoid CB1 Receptor. Med Res Rev..

[19] Khurana L, Mackie K, Piomelli D, Kendall DA. Modulation of CB1 cannabinoid receptor by allosteric ligands: pharmacology and therapeutic opportunities. Neuropharmacology. 124;2017:3–12. 10.1016/j.neuropharm.2017.05.018 PMC5540789 28527758

[20] Ignatowska-Jankowska BM, Baillie GL, Kinsey S, Crowe M, Ghosh S, Owens RA (2015). A cannabinoid CB 1 receptor-positive allosteric modulator reduces neuropathic pain in the mouse with no psychoactive effects. Neuropsychopharmacology..

[21] Alaverdashvili M, Laprairie RB (2018). The future of type 1 cannabinoid receptor allosteric ligands. Drug Metab Rev.

[22] Nguyen T, Thomas BF, Zhang Y (2019). Overcoming the psychiatric side effects of the cannabinoid CB1 receptor antagonists: current approaches for therapeutics development. Curr Top Med Chem.

[23] Efimova EV, Gainetdinov RR, Budygin EA, Sotnikova TD (2016). Dopamine transporter mutant animals: a translational perspective. J Neurogenet..

[24] Giros B, Jaber M, Jones SR, Wightman RM, Caron MG (1996). Hyperlocomotion and indifference to cocaine and amphetamine in mice lacking the dopamine transporter. Nature..

[25] Ferris MJ, Milenkovic M, Liu S, Mielnik CA, Beerepoot P, John CE (2014). Sustained N-methyl-d-aspartate receptor hypofunction remodels the dopamine system and impairs phasic signaling. Eur J Neurosci.

[26] Mohn AR, Gainetdinov RR, Caron MG, Koller BH (1999). Mice with reduced NMDA receptor expression display behaviors related to schizophrenia. Cell..

[27] Ramsey AJ (2009). NR1 knockdown mice as a representative model of the glutamate hypothesis of schizophrenia. Prog Brain Res.

[28] Milenkovic M, Mielnik CA, Ramsey AJ (2014). NMDA receptor-deficient mice display sexual dimorphism in the onset and severity of behavioural abnormalities. Genes Brain Behav.

[29] Duncan GE, Moy SS, Perez A, Eddy DM, Zinzow WM, Lieberman JA (2004). Deficits in sensorimotor gating and tests of social behavior in a genetic model of reduced NMDA receptor function. Behav Brain Res.

[30] Islam R, Trépanier M-O, Milenkovic M, Horsfall W, Salahpour A, Bazinet RP (2017). Vulnerability to omega-3 deprivation in a mouse model of NMDA receptor. NPJ Schizophr.

[31] Ralph RJ, Paulus MP, Fumagalli F, Caron MG, Geyer MA (2001). Prepulse inhibition deficits and perseverative motor patterns in dopamine transporter knock-out mice: differential effects of D1 and D2 receptor antagonists. J Neurosci.

[32] Stiefl N, Gedeck P, Chin D, Hunt P, Lindvall M, Spiegel K (2015). FOCUS-Development of a global communication and modeling platform for applied and computational medicinal chemists. J Chem Inf Model.

[33] Cawston EE, Hunter MR, Glass M. Allosteric modulation of the cannabinoid CB1 receptor. In: Preedy VR, editor. Handbook of cannabis and related pathologies: biology, pharmacology, diagnosis, and treatment. Vol. 68. United Kingdom: Academic Press; 2017. p. 573–83.

[34] Hunter MR, Finlay DB, Macdonald CE, Cawston EE, Grimsey NL, Glass M. Real-time measurement of cannabinoid receptor-mediated cAMP signaling. Methods Enzymol. 2017;593:43–59. 10.1016/bs.mie.2017.05.001 28750814

[35] Long LE, Chesworth R, Huang X-FF, McGregor IS, Arnold JC, Karl T (2010). A behavioural comparison of acute and chronic 9- tetrahydrocannabinol and cannabidiol in C57BL/6JArc mice. Int J Neuropsychopharmacol..

[36] Mielnik CA, Horsfall W, Ramsey AJ (2014). Diazepam improves aspects of social behaviour and neuron activation in NMDA receptor-deficient mice. Genes Brain Behav.

[37] Moy SS, Nadler JJ, Young NB, Perez A, Holloway LP, Barbaro RP (2007). Mouse behavioral tasks relevant to autism: Phenotypes of 10 inbred strains. Behav Brain Res.

[38] Cawston EE, Redmond WJ, Breen CM, Grimsey NL, Connor M, Glass M (2013). Real-time characterization of cannabinoid receptor 1 (CB1) allosteric modulators reveals novel mechanism of action. Br J Pharm.

[39] Moreira FA, Crippa JAS (2009). The psychiatric side-effects of rimonabant. Rev Bras Psiquiatr.

[40] O’Brien LD, Wills KL, Segsworth B, Dashney B, Rock EM, Limebeer CL (2013). Effect of chronic exposure to rimonabant and phytocannabinoids on anxiety-like behavior and saccharin palatability. Pharm Biochem Behav.

[41] Wadenberg MLG, Hicks PB (1999). The conditioned avoidance response test re-evaluated: is it a sensitive test for the detection of potentially atypical antipsychotics?. Neurosci Biobehav Rev.

[42] Wadenberg MLG (2009). Conditioned avoidance response in the development of new antipsychotics. Curr Pharm Des.

[43] Ross RA, Brockie HC, Stevenson LA, Murphy VL, Templeton F, Makriyannis A (1999). Agonist-inverse agonist characterization at CB1 and CB2 cannabinoid receptors of L759633, L759656 and AM630. Br J Pharm.

[44] Christopoulos A, Kenakin T (2002). G protein-coupled receptor allosterism and complexing. Pharmacol Rev..

[45] Steindel F, Lerner R, Häring M, Ruehle S, Marsicano G, Lutz B (2013). Neuron-type specific cannabinoid-mediated G protein signalling in mouse hippocampus. J Neurochem.

[46] Busquets-Garcia A, Bains J, Marsicano G (2018). CB 1 receptor signaling in the brain: extracting specificity from ubiquity. Neuropsychopharmacology..

[47] Kruk-Slomka M, Banaszkiewicz I, Slomka T, Biala G (2019). Effects of fatty acid amide hydrolase inhibitors acute administration on the positive and cognitive symptoms of schizophrenia in mice. Mol Neurobiol.

[48] Stornaiuolo M, Bruno A, Botta L, Regina G La, Cosconati S, Silvestri R, et al. Endogenous vs exogenous allosteric modulators in GPCRs: a dispute for shuttling CB1 among different membrane microenvironments. Sci Rep. 2015;5. 10.1038/srep15453. 10.1038/srep15453 PMC4612305 26482099

[49] Van Den Buuse M (2010). Modeling the positive symptoms of schizophrenia in genetically modified mice: Pharmacology and methodology aspects. Schizophr Bull..

[50] Wang SM, Han C, Lee SJ, Jun TY, Patkar AA, Masand PS (2017). Investigational dopamine antagonists for the treatment of schizophrenia. Expert Opin Investig Drugs.

[51] Takahashi H, Hashimoto R, Iwase M, Ishii R, Kamio Y, Takeda M (2011). Prepulse inhibition of startle response: recent advances in human studies of psychiatric disease. Clin Psychopharmacol Neurosci.

[52] Varvel SA, Anum EA, Lichtman AH (2005). Disruption of CB1 receptor signaling impairs extinction of spatial memory in mice. Psychopharmacology (Berl).

[53] Reibaud M, Obinu MC, Ledent C, Parmentier M, Böhme GA, Imperato A. Enhancement of memory in cannabinoid CB1 receptor knock-out mice. Eur J Pharmacol. 1999;379. 10.1016/S0014-2999(99)00496-3. 10.1016/s0014-2999(99)00496-3 10499380

[54] Li M, He W, Mead A (2009). An investigation of the behavioral mechanisms of antipsychotic action using a drug-drug conditioning paradigm. Behav Pharm.

[55] Gobira PH, Ropke J, Aguiar DC, Crippa JAS, Moreira FA (2013). Animal models for predicting the efficacy and side effects of antipsychotic drugs. Rev Bras Psiquiatr.

[56] Aso E, Andrés-Benito P, Ferrer I (2018). Genetic deletion of CB1 cannabinoid receptors exacerbates the Alzheimer-like symptoms in a transgenic animal model. Biochem Pharm.

[57] Rowley S, Sun X, Lima IV, Tavenier A, de Oliveira ACP, Dey SK (2017). Cannabinoid receptor 1/2 double-knockout mice develop epilepsy. Epilepsia..

[58] Lane DA, Chan J, Lupica CR, Pickel VM (2010). Cannabinoid-1 receptor gene deletion has a compartment-specific affect on the dendritic and axonal availability of μ-opioid receptors and on dopamine axons in the mouse nucleus accumbens. Synapse..

[59] Hungud BL, Szakall I, Adam A, Basavarajappa BS, Vadasz C (2003). Cannabinoid CB1 receptor knockout mice exhibit markedly reduced voluntary alcohol consumption and lack alcohol-induced dopamine release in the nucleus accumbens. J Neurochem.

[60] Vallée M, Vitiello S, Bellocchio L, Heb́ert-Chatelain E, Monlezun S, Martin-Garcia E (2014). Pregnenolone can protect the brain from cannabis intoxication. Science (80-).

