# Supplementary material for: A novel allosteric modulator of the cannabinoid CB1 receptor ameliorates hyperdopaminergia endophenotypes in rodent models
Source: Neuropsychopharmacology. 2020 Oct 8;46(2):413–22. doi: 10.1038/s41386-020-00876-5 (PMC7852560; doi:10.1038/s41386-020-00876-5)
Supplement: Supplementary file 1 — Mielniketal2020_Supplemental Material [file 41386_2020_876_MOESM1_ESM.docx]

**Supplementary Information**

**A Novel Allosteric Modulator of the Cannabinoid CB_1_** **Receptor Ameliorates Hyperdopaminergia Endophenotypes in Rodent Models**

Catharine A. Mielnik^1^, Kim S. Sugamori^1^, David B. Finlay^2^, Hayley H.A. Thorpe^3^, Matthieu Schapira^4^, Nirunthan Sivananthan^1^, Chun Kit Li^1^, Vincent M. Lam^1^, Sean Harrington^1^, Mostafa H. Abdelrahman^5^, Laurent A. Trembleau^5^, W. McIntyre Burnham^1^, Jibran Y Khokhar^3^, Ali Salahpour^1^, Amy J. Ramsey^1^, Michelle Glass^2^, Iain R. Greig^5^, Ruth A. Ross^1^*

**METHODS AND MATERIALS**

## **Synthesis of ABM300 (5-(5-Chloro-3-ethyl-1H-indol-2-yl)-N-phenyl-1,3,4-oxadiazol-2-amine)**

## To a solution of isothiocyanatobenzene (14.36 mmol) in ethanol (25 mL), 60% hydrazine monohydrate (0.96 ml, 17.23 mmol) was added and the mixture was stirred at room temperature for 30 min. The precipitate formed was filtered off, washed with ethanol to provide N-phenylhydrazinecarbothioamide as a white solid. ^1^H NMR (400 MHz, DMSO-d_6_) δ 9.66 (s, 1H, NHCSNHNH_2_), 9.13 (s, 1H, NHCSNHNH_2_), 7.65 (d, J = 8.0 Hz, 2H, Ar-H), 7.30 (t, J = 7.8 Hz, 2H, Ar-H), 7.09 (t, J = 7.4 Hz, 1H, Ar-H), 4.80 (s, 2H, NHCSNHNH_2_). ^13^C NMR (101 MHz, DMSO-d_6_) δ 179.38, 139.24, 128.08, 124.14, 123.54.

## A mixture of 5-chloro-3-ethyl-1H-indole-2-carboxylic acid (0.894 mmol, 1 eq), N-phenylhydrazinecarbothioamide (1.073 mmol, 1.2 eq) and EDC (0.51 g, 2.682 mmol, 3 eq) in DCM (25 mL) was stirred at room temperature overnight. The reaction mixture was purified by silica gel column chromatography (EtOAc). After removal of the solvent, the residue was suspended in few mL of Et_2_O, the precipitate filtered and washed with Et_2_O to give 5-(5-chloro-3-ethyl-1H-indol-2-yl)-N-phenyl-1,3,4-oxadiazol-2-amine as a pale yellow solid. ^1^H NMR (400 MHz, DMSO-d_6_) δ 11.88 (s, 1H, indole NH), 10.64 (s, 1H, NH), 7.70 (s, 1H, Ar-H), 7.63 (d, J = 7.8 Hz, 2H, Ar-H), 7.45 – 7.32 (m, 3H, Ar-H), 7.20 (d, J = 8.7 Hz, 1H, Ar-H), 7.02 (t, J = 7.3 Hz, 1H, Ar-H), 3.04 (q, J = 7.5 Hz, 2H, CH_2_CH_3_), 1.23 (t, J = 7.4 Hz, 3H, CH_2_CH_3_). ^13^C NMR (101 MHz, DMSO-d_6_) δ 160.01, 153.77, 139.08, 135.85, 129.56, 128.66, 124.50, 124.23, 122.42, 119.95, 119.56, 119.06, 117.63, 114.01, 17.59, 16.06. HRESI-MS m/z calcd for [M+H]^+^ C_18_H_16_ClN_4_O: 339.1007, found: 339.1010. mp 252-254°C.

***In vitro* metabolic stability assays: human and rat liver microsomal stability**

The study of drug stability in the presence of liver microsomes is accepted as a valuable model for permitting rapid prediction of *in vivo* drug stability (see Kerns & Di, 2008). Microsomal stability studies were performed by Cyprotex Ltd (Macclesfield, UK). The *in vitro* metabolic stability of ABM300 was measured in the presence of human or rat liver microsomes by determination of the rate of compound disappearance. Microsomes (final protein concentration 0.5 mg/mL), 0.1 M phosphate buffer pH 7.4, and test compound (final concentration 3 μM; diluted from 10 mM stock solution to give a final DMSO concentration of 0.25%) were incubated at 37°C prior to the addition of NADPH (final concentration 1 mM) to initiate the reaction with a final incubation volume of 25 µL. A control incubation was included for each compound tested, where 0.1 M phosphate buffer pH 7.4 was added instead of NADPH. The control compounds testosterone and 7‑hydroxycoumarin were included in each experiment and all incubations were performed singularly for each compound. Each compound was incubated for 0, 5, 15, 30, and 45 minutes with the control (minus NADPH) incubated for 45 minutes only. The reactions were terminated by the addition of 50 µL methanol containing internal standard. The incubation plates were centrifuged at 2500 rpm for 20 minutes at 4°C to precipitate protein. Following protein precipitation, the sample supernatants were combined in cassettes of up to 4 compounds and analyzed using standard LC-MS/MS conditions. From a plot of the natural logarithm of the peak area ratio (i.e., the ratio of compound peak area: internal standard peak area) against time, the gradient of the line was determined. Subsequently, half-life and intrinsic clearance were calculated using the equations below:

Eliminated rate constant (k) = (- gradient).

Half-life (t_1/2_) (min) = 0.693 / k.

Intrinsic Clearance (CL_int_) (µL/min/million cells) = (V x 0.693) / t_1/2_.

wherein V = Incubation volume (µL/mg microsomal protein).

***In vivo* metabolic stability and distribution in a mouse model**

Single dose *in vivo* PK studies were conducted by Sai Life Ltd (Pune, India) to investigate the plasma pharmacokinetics and brain distribution of ABM300 in male C57BL6 mice. Twenty-four male C57BL6 mice were administered intraperitoneally with 10 mg/kg ABM300 prepared in 5% NMP, 5% Solutol HS-15, and 90% normal saline. Blood samples (approximately 60 μL; n=3 mice per time-point) were taken at 0.08, 0.25, 0.5, 1, 2, 4, 6 and 8 hrs. Immediately after collection of blood, brain samples were collected from the set of three mice at each time point. Plasma samples were separated by centrifugation of whole blood, while brain samples were homogenized in ice-cold phosphate buffer saline (pH 7.4). Total homogenate volume was three times the brain weight. Plasma and homogenates were stored below -70±10 °C until analysis. The extraction procedure for plasma/brain samples and the spiked plasma/brain calibration standards were identical. To a 25 µL volume of study sample (dilution factor applied to some samples) or spiked plasma/brain calibration standard, 100 µL of internal standard prepared in acetonitrile (Glipizide, 500 ng/mL) was added, except for blank where 100 µL of acetonitrile was added. Samples were vortexed for 5 minutes, centrifuged for 10 minutes at 4000 rpm at 4 °C and 100 µL of clear supernatant was analyzed with fit-for-purpose C-MS/MS method (LLOQ- 1.02 ng/mL for plasma and 3.06 ng/g for brain). Pharmacokinetic parameters were calculated using the non-compartmental analysis tool of Phoenix WinNonlin® (Version 7.0). Areas under the concentration time curve (AUC) values were calculated by linear trapezoidal rule. The elimination rate constant, K_e_, was determined by regression analysis of the linear terminal portion of the log plasma concentration-time curve using GraphPad Prism 8.0 and the equation t_1/2_ = 0.693/K_e_.

All procedures of the study were conducted in accordance with the guidelines provided by the Committee for the Purpose of Control and Supervision of Experiments on Animals (CPCSEA) as published in The Gazette of India, December 15, 1998. Prior approval of the Institutional Animal Ethics Committee (IAEC) was obtained before initiation of the study. This study was performed with approval of Institutional Animal Ethics Committee (IAEC) in accordance with requirement of the CPCSEA, India.

**Predictive Model of ABM300 bound to CB_1_R**

The crystal structure of the CB_1_R-CP55940-ORG27569 complex (PDB code 6kqi) was loaded into ICM (Molsoft, San Diego), hydrogens were added, and rotameric states of hydroxy groups, histidine, asparagine and glutamine side-chains optimized. ICM’s ligand editor was used to strip ORG27569 to the indole core scaffold shared with ABM300, and to incrementally grow the scaffold into ABM300, with a Monte Carlo-based energy minimization in the internal coordinate space at each step (Stiefl et al., 2015).

**SafetyScreen44 Panel Assay**

Potential off-target effects of ABM300 were assessed in the Eurofins Cerep SafetyScreen44 Panel Assay (Celle-Lévescault, France). ABM300 was screened at a 1 µM concentration in duplicate to measure interference with the binding of native ligands at 44 different receptors, ion channels and enzymes. For receptor assays, binding was calculated as percent inhibition of the binding of a radioactively labeled ligand specific for each target. For enzyme assays, the inhibition effect was calculated as percent inhibition of control enzyme activity. Tests were performed in duplicate, and results are averages of those two tests. See <https://www.eurofinsdiscoveryservices.com>.

**Cell Culture**

The human CB_1_R (hCB_1_R) was stably transfected into HEK293 (E. E. Cawston, Hunter, & Glass, 2017) and CHO-K1 cells (Ross et al., 1999). The hCB_1_R CHO cells were cultured in DMEM/F12 supplemented with 10% FBS, 600µg/mL G418, and 300µg/mL hygromycin. The hCB_1_R HEK293 cells were cultured in DMEM supplemented with 10% FBS and 250μg/mL Zeocin. All cells were maintained at 37°C, 5% CO_2_ in a humidified atmosphere.

## **Equilibrium Binding Assays**

Binding assays in hCB_1_R CHO cells were performed by Eurofins Cerep (Celle-Lévescault, France) with the CB_1_R agonist, [^3^H]CP55,940 (0.5 nM), which had a Kd of 3.5 nM in these cells. Assays were carried out at 37°C for 120 min before termination by the addition of ice-cold wash buffer and vacuum filtration. Radioactivity was quantitated by liquid scintillation spectrometry. Specific binding was defined as the difference between the binding that occurred in the presence and absence of 10 µM of WIN55212. Data were analyzed by nonlinear regression analysis of allosteric modulator titration model using GraphPad Prism 8.0 (GraphPad, San Diego, CA). In order to fit the data of the allosteric modulator titration model, the HotKDnm and RadioligandNM parameters were set to 3.5 and 0.5 respectively. The results of this analysis are presented as *E_max_* with 95% confidence limits, pK_B_ (logK_B_) ± SEM and α ± SEM.

**PathHunter® Beta-arrestin Assays**

The PathHunter® β-Arrestin assay was conducted by Eurofins Pharma Discovery Services. The assay monitors the activation of a GPCR in a homogenous, non-imaging assay format using a technology developed by DiscoverX called Enzyme Fragment Complementation (EFC) with β-galactosidase (β-Gal) as the functional reporter. Vehicle concentration was 1% DMSO in these assays. See <https://www.eurofinsdiscoveryservices.com>

**ERK1/2 Phosphorylation Assay**

CB_1_R-mediated ERK1/2 MAP-kinase phosphorylation was quantified using an AlphaLISA® Surefire® Ultra™ pERK1/2 Assay (PerkinElmer, Woodbridge, ON). CHO-K1 cells stably expressing the human CB_1_R were grown in DMEM/F12 medium containing 10% FBS, 300 ug/ml hygromycin B, 600 μg/ml G418 and 1 x penicillin-streptomycin at 37 °C, 5% CO_2_. hCB_1_R cells were dissociated with a non-enzymatic dissociation solution (ThermoFisher Scientific, Burlington, ON) and plated in 96 well plates at a density of 40,000 cells/well. The following day, hCB_1_R cells were serum-starved for 4 hours with DMEM/F12 medium containing 0.1% BSA but no phenol red, unless otherwise indicated. Cells were pretreated with increasing concentrations of ABM300 (10^-10^ – 10^-5^ M) for 30 min at 37 °C, 5% CO_2_ prior to addition of 40 nM CP55,940 (Millipore Sigma, St. Louis, MO) and incubation for 8 min at 37 °C, 5% CO_2_. Vehicle concentration was 1% DMSO, 0.1% BSA. Alternatively, the effect of pre-incubation with fixed concentrations of ABM300 (100 nM, 1000 nM) on either CP55,940 (10^-10^ – 10^-5^ M) or AEA (10^-9^ – 10^-4^ M) stimulated ERK1/2 phosphorylation were assessed as described, but without 0.1% BSA. Reactions were stopped by the addition of 50 μL ice-cold lysis buffer, and cells were lysed by shaking on an orbital shaker for 10 min at room temperature. Plates were stored at -80 °C prior to performing the Surefire Ultra assay with 5 μL lysate, 2.5 μL acceptor beads and 2.5 μL donor beads in 384-well white Proxiplates as per the manufacturer’s instructions. Plates were incubated in the dark for 3 hr at room temperature and were read with a BioTek Synergy Neo plate-reader (BioTek Instruments, Winooski, VT) using AlphaScreen settings. Results are presented as the percent stimulation of ERK1/2 phosphorylation by CP55,940 alone. Data were analyzed by non-linear regression analysis of the log CP55,940 concentration versus response using GraphPad Prism 8.0 (GraphPad, San Diego, CA).

**Cyclic AMP Assays**

Cyclic AMP accumulation was measured using a DiscoverX HitHunter® cAMP Assay for Small Molecules (DiscoverX, Fremont, CA). hCB_1_R CHO cells were seeded at a density of 40,000 cells/well in 96-well white plates and grown overnight at 37 °C, 5% CO_2_ in a humidified incubator. The following day, cell media was replaced with 30 μL cAMP assay buffer (HBSS containing 10 mM HEPES, 0.1% BSA and 100 μM IBMX) for 60 min, followed by addition of 7.5 μL of a 6X concentration of ABM300 (final concentrations of 10^-10^ – 10^-5^ M) and incubation for 30 min at 37°C, 5% CO_2_. Cells were subsequently treated with the EC_80_ concentration of CP55,940 (final concentration of 40 nM) and 6X forskolin (final concentration of 5 μM) and incubated for 30 min at 37 °C, 5% CO_2._  Vehicle concentration was 1% DMSO, 0.1% BSA. Following agonist incubation, 15 μl of cAMP antibody reagent and 60 μl of working cAMP detection solution (containing 19 parts cAMP lysis buffer, 5 parts Substrate Reagent 1, 1 part Substrate Reagent 2 and 25 parts of cAMP Solution D) were added to all wells. Assay plates were incubated in the dark for 1 hr at room temperature, followed by addition of 60 μl cAMP solution A to all wells and incubation for 3 hr at room temperature in the dark. Plates were read using a GloMax Discover plate-reader (Promega, Madison, WI). Results were calculated as the percent inhibition of the CP55,940 mediated response and were analyzed by non-linear regression analysis of sigmoidal dose-response curves using GraphPad Prism 8.0.

The cAMP BRET experiments were performed as previously described (Erin E. Cawston et al., 2013; Hunter et al., 2017). hCB_1_R HEK293 cells were seeded in 100 mm dishes in normal culture medium. One day after plating, the cells were approximately 50% confluent, and were transfected with 5 µg of the CAMYEL biosensor using linear polyethylenimine (in a 1:6 ratio of DNA:PEI). After 24 hr, the cells were lifted with 0.05% trypsin/EDTA and plated in normal culture medium into white 96 well CulturPlates (PerkinElmer) which had been pre-coated with 0.05 mg/ml poly-D-lysine, at a density of 60,000 cells/well. After culture overnight, plate wells were handled for one run at time. Culture medium was removed by aspiration, and wells were washed once with PBS and then left in 60 µL/well of phenol red-free, high glucose DMEM, supplemented with 10 mM HEPES (pH 7.4) and 0.1% BSA (“assay medium”). The assay plate was then returned to the incubator for 30 min equilibration prior to stimulation. All drugs were made up at 10x the desired concentration in assay medium (ethanol and DMSO vehicles were load-matched in all wells). Drug dilutions were then combined in equal volumes in low lipid binding, polypropylene V-well dispensing plates (Hangzhou Gene Era Biotech Co Ltd, Zhejiang, China). Coelenterazine H (Nanolight, Pinetop, AZ; prepared at 5 mM in absolute ethanol) was diluted in assay medium at 10x concentration immediately prior to dispensing. At the end of the 30 min equilibration period, 10 µL of coelenterazine H dilution was dispensed per well (final concentration of 5 µM), and the assay plate was then incubated for 5 minutes in a pre-warmed, 37 °C LUMIstar Omega plate reader (BMG Labtech, Ortenburg, Germany). The drug mixture was then dispensed to the assay plate using a multichannel pipette (30 µL/well; giving a final stimulation volume of 100 µL/well) and the plate reader was immediately initiated. Reads used simultaneous BRET1 filters (475-30 and 353-30 nm), and cycles were set to run for approximately 20 min.

**Animals**Animal housing and experimentation were carried out in accordance with the Canadian Council in Animal Care (CCAC) guidelines for the care and use of animals and following protocols approved by the Faculty of Medicine and Pharmacy Animal Care Committee at the University of Toronto and the University of Guelph Animal Care Committee, respectively. For the genetic models of hyperdopaminergia, mice were group housed with littermates on a 12-h light-dark cycle (0700 to 1900h) and were given *ad libitum* access to food (2018 Teklad Global 18% Protein Rodent Diet, Envigo, Madison WI, www.envigo.com). Tail biopsy for genotyping and toe tattooing for identification was performed at PD13 (±3 days) and mice were weaned at P21. Behavioural and biochemical tests performed in adult mice (PD>70) and balanced for sex (equal numbers of males and females in each cohort, as best possible).

**Murine Models of Psychosis**

GluN1-knockdown (GluN1KD and wildtype, WT, littermates) mice used in this study were F1 progeny of a defined genetic background (GluN1KD heterozygotes C57Bl/6J x GluN1KD heterozygotes 129/SvlmJ). The knockdown was generated by targeted insertion of a neomycin cassette into intron 19 of the *Grin1* sequence, as described in (Mohn, Gainetdinov, Caron, & Koller, 1999). Dopamine transporter knockout (DATKO and WT littermates) mice used in this study were on a C57Bl/6J background. The knockout was generated via homologous recombination, leading to no DAT mRNA being expressed, as described by (Giros, Jaber, Jones, Wightman, & Caron, 1996).

**Anxiety Behavior Testing - Elevated Plus Maze (EPM)**

Anxiety behavior was assessed in C57Bl/6J mice (male and female; PD>70) via EPM (Moy et al., 2007), comparing vehicle (1:1:18 vehicle, 95% ethanol:Tween80:0.9% NaCl), rimonabant (10 mg/kg), and ABM300 (10 mg/kg), given 30-min. (i.p.) before testing. The EPM was composed of 4 opaque-white arms (2 opposite arms closed, 2 opposite arms open), arranged in a plus shape, with an open center. The dimensions were as follows; maze elevation (38.7cm), open arm (L:30.5 cm, W:5 cm, H:0 cm), closed arm (L:30.5 cm, W:5 cm, H:15.2 cm) and center (5 cm x 5 cm). The experiment mouse was placed in the center of the maze and allowed to freely explore the maze for 8-min (50lux), while being tracked with an overhead camera. Open and closed arm times were recorded and collected by Biobserve Viewer3 software. The percentage of time spent in the open arms as compared to closed arms and center time was calculated and expressed as a percent of time spent in the open or closed arms of the maze.

**Cannabinoid-Induced Tetrad Behaviors**

Male C57Bl/6J mice (PD>70) were tested on the tetrad, as previously described (Long et al., 2010), comparing vehicle (as previously described above), Δ^9^-tetrahydrocannabinol (THC; 10 mg/kg; gift from MedReleaf, Markham, ON) and ABM300 (10 mg/kg), given 30-min. (i.p.) before testing. The tetrad was performed (1) catalepsy, (2) locomotion, (3) temperature, and (4) analgesia. Catalepsy: time (s) spent immobile with front paws on a plastic rod (1.5cm diameter), at a height of 4.5-cm from the table, before adjusting abnormal posture (either front paws being removed from the bar, or back paws climbing onto bar). Locomotion: horizontal activity (cm) was immediately measured in an open field test, following catalepsy, for 15-min. in 1-min bins. Temperature: rectal temperature (°C) was measured immediately following open-field testing, using a thermometer with rectal probe (Labcorp, Fisher, Waltham, MA). Analgesia: latency (s) to withdraw tail from hot water bath (52 °C), to assess tail-flick antinociception, was completed immediately following temperature readings.

**RNAscope Visualization and Quantification of *Cnr1***

Expression of *Cnr1* mRNA in WT and mutant (GluN1KD and DATKO) mice was visualized by fluorescent *in situ* hybridization largely following the RNAscope Multiplex Fluorescent Reagent Kit protocol (ACD Bio, Newark, CA). Mice were euthanized via cervical dislocation, and brains removed, frozen in dry-ice chilled 2-methylbutane (Millipore Sigma), and stored at -80 °C. Frozen mouse brains were processed with a cryostat to produce 20 µm sagittal sections at ~1.5 mm from midline that were mounted onto SuperFrost Plus charged slides (Fisher Scientific). Sections were fixed by submersion in 4% paraformaldehyde, dehydrated in ethanol, and treated with RNAscope Protease IV. Sections were hybridized to a *Cnr1* probe (product reference ID 420721-C2 ACDBio) or to control probe mixtures (positive control probes reference ID 320881, negative control probes reference ID 320871), and counter-stained with DAPI. Processed slides were stored at 4 °C overnight before imaging at 20X magnification with a Zeiss LSM 880 Super Resolution Confocal (Zeiss, Oberkochen, DEU). Quantification of probe hybridization was completed by measuring total mean pixel intensity in ImageJ, via 8-bit images at 2048x2048 frame size.

**Conditioned Avoidance Response (CAR) in Sprague Dawley Rats**

Male Sprague Dawley rats (PD>63) were trained on a CAR protocol and responding following drug exposure was determined. Drug was administered daily, via i.p. injection, 30-min. prior to behavioural testing. Rats were treated with either vehicle (same as previously described, 2 mL/kg), ABM300 (10 mg/kg) or olanzapine (OLA; 1 mg/kg). The CAR protocol was completed over 22-24 days: acquisition (10-12 days), vehicle baseline (3 days), drug exposure (OLA vs. ABM300; 7 days), and re-emergence (vehicle; 2 days) (Li, Fletcher, & Kapur, 2007). Animals were trained to learn a house light (CS) as predictive of an avoidable 0.6mA scrambled foot shock (US). Each session consisted of 50 trials and animals avoided >70% of trials on the final acquisition session were tested for CAR during vehicle baseline, drug exposure, and re-emergence. In addition to CAR testing, both temperature and catalepsy were recorded in these animals (measure of potential CB_1_R agonism). Temperature: recorded on first day of drug exposure, prior to injection and 60-90-min. following drug exposure. Catalepsy: recorded on third day of drug exposure, prior to injection and 25-min. following injection.

**Drug Administration (Genetic Models of Hyperdopaminergia)**

The acute effect of a novel CB_1_R negative allosteric modulator, ABM300, were investigated in wildtype and genetic models. The drug was administered at 10 mg/kg in 1:1:18 vehicle (95% ethanol : Tween80 : 0.9% NaCl), via intraperitoneal (i.p.) injection 30-min. prior to behavioural or biochemical testing. The effect of ABM300 was compared to olanzapine (OLA; atypical antipsychotic, 1 mg/kg; Millipore Sigma, Cat.# O1141, Toronto, ON). For all behavioural and biochemical tests, drugs were dissolved in the previously described vehicle, and injected (10 ml/kg) 30-min. prior to testing or sacrifice, unless otherwise specified.

**Behavioral Testing (Genetic Models of Hyperdopaminergia)**

All behavioral tests were completed between 09:00 and 15:00h. All mice were tested as follows: Day 1 – open field test, and Day 3 – pre-pulse inhibition.

*Open Field Test (OF):* Novelty-induced locomotor activity, stereotypy, and vertical activity were measured as previously described (Islam et al., 2017; Mielnik, Horsfall, & Ramsey, 2014; Milenkovic, Mielnik, & Ramsey, 2014) using digital activity monitors (Omnitech Electronics, Columbus, OH, USA) on the first day of testing. 30-min. following injection, mice were placed in novel Plexiglas arenas (20 x 20 x 45 cm) and their locomotor and stereotypic activity were recorded over a 120-min. period in dim light (15-16lux). Activity was tracked via infrared light beam sensors; total distance traveled and stereotypic movements were collected in 5-min. bins.

*Pre-Pulse Inhibition (PPI):* PPI of the acoustic startle response was measured on day 3, via SR-LAB equipment and software from San Diego Instruments. Accelerometers were calibrated to 700±5 mV and output voltages were amplified and analyzed for voltage changes using SR Analysis (San Diego Instruments, San Diego, CA, USA), and exported as an excel file. Background white noise was maintained at 65dB. PPI was measured in a 30-min test with 80 randomized trials of: (1) 10 trials pulse alone (2) 10 trials pre-pulse alone (for each pre-pulse), (3) 10 trials pre-pulse plus pulse (for each pre-pulse), and (4) 10 trials no pulse. 5 pulse alone trials were performed before and after the 80 trials, totaling 90 trials per run. The pre-pulse (4dB, 8dB, or 16dB) was presented 100ms prior to the startle pulse (165dB). The inter-stimulus interval (ISI) was randomized between 5 and 20s. Experimental mice were placed in a cylindrical tube on a platform in a soundproof chamber. Mice were allowed to acclimatize in the chamber and to the background noise for 300s, followed by 5 consecutive pulse alone trials, then by 80 randomized trials (as described above) and then 5 consecutive pulse alone trials. Pre-pulse inhibition was measured as a decrease in the amplitude of startle response to a 100dB acoustic startle pulse, following each pre-pulse (4dB, 8dB and 16dB).

**Quantification and Statistical Analysis**

Statistical parameters, the definition of measures and statistical significance are reported in the figures and the figure legends. Data are represented as mean ± SEM or SD, as indicated in figure legends. Sample number (n), indicating independent biological samples (balanced for sex *in vivo* unless otherwise indicated, no effects of sex unless otherwise stated), are indicated in each figure and/or figure legend. Statistically significant outliers were calculated and excluded, using the Grubb’s Test. Data were analyzed either using a one- or two-way ANOVA (repeated measures) where indicated, with multiple comparisons and post-hoc Sidak’s test, as indicated in figure legends. CAR data was analyzed using a RM ANOVA with Bonferroni correction to determine within group differences between baseline day 3 and drug testing data, and vehicle baseline data used as covariates to determine main effects, interactions and between group differences. Data analysis was not blinded. Differences in means were considered statistically significant at p<0.05. Significance levels are as follows; *p<0.05, **p<0.01, ***p<0.001, ****p<0.0001, ns – not significant. All data analyses were performed using the Graphpad Prism 8.0 software and/or IBM SPSS 23.0 Software, unless otherwise indicated.

**REFERENCES**

Cawston, E. E., Hunter, M. R., & Glass, M. (2017). Allosteric Modulation of the Cannabinoid CB1 Receptor. In *Handbook of Cannabis and Related Pathologies: Biology, Pharmacology, Diagnosis, and Treatment* (Vol. 68, pp. 573–583). https://doi.org/10.1016/B978-0-12-800756-3.00070-3

Cawston, Erin E., Redmond, W. J., Breen, C. M., Grimsey, N. L., Connor, M., & Glass, M. (2013). Real-time characterization of cannabinoid receptor 1 (CB1) allosteric modulators reveals novel mechanism of action. *British Journal of Pharmacology*, *170*(4), 893–907. https://doi.org/10.1111/bph.12329

Giros, B., Jaber, M., Jones, S. R., Wightman, R. M., & Caron, M. G. (1996). Hyperlocomotion and indifference to cocaine and amphetamine in mice lacking the dopamine transporter. *Nature*, *379*(6566), 606–612. https://doi.org/10.1038/379606a0

Hunter, M. R., Finlay, D. B., Macdonald, C. E., Cawston, E. E., Grimsey, N. L., & Glass, M. (2017). Real-Time Measurement of Cannabinoid Receptor-Mediated cAMP Signaling. In *Methods in Enzymology* (Vol. 593, pp. 43–59). Academic Press Inc. https://doi.org/10.1016/bs.mie.2017.05.001

Islam, R., Trépanier, M.-O., Milenkovic, M., Horsfall, W., Salahpour, A., Bazinet, R. P., & Ramsey, A. J. (2017). Vulnerability to omega-3 deprivation in a mouse model of NMDA receptor. *Npj Schizophrenia*, *3*(1), 12. https://doi.org/10.1038/s41537-017-0014-8

Kerns, E. H., & Di, L. (2008). *Drug-like Properties: Concepts, Structure Design and Methods*. *Drug-like Properties: Concepts, Structure Design and Methods*. Elsevier Inc.

Li, M., Fletcher, P. J., & Kapur, S. (2007). Time course of the antipsychotic effect and the underlying behavioral mechanisms. *Neuropsychopharmacology*, *32*(2), 263–272. https://doi.org/10.1038/sj.npp.1301110

Mielnik, C. A. A., Horsfall, W., & Ramsey, A. J. J. (2014). Diazepam improves aspects of social behaviour and neuron activation in NMDA receptor-deficient mice. *Genes, Brain and Behavior*, *13*(7), 592–602. https://doi.org/10.1111/gbb.12155

Milenkovic, M., Mielnik, C. A. A., & Ramsey, A. J. J. (2014). NMDA receptor-deficient mice display sexual dimorphism in the onset and severity of behavioural abnormalities. *Genes, Brain and Behavior*, *13*(8), 850–862. https://doi.org/10.1111/gbb.12183

Mohn, A. R., Gainetdinov, R. R., Caron, M. G., & Koller, B. H. (1999). Mice with reduced NMDA receptor expression display behaviors related to schizophrenia. *Cell*, *98*(4), 427–436. https://doi.org/10.1016/S0092-8674(00)81972-8

Ross, R. A., Brockie, H. C., Stevenson, L. A., Murphy, V. L., Templeton, F., Makriyannis, A., & Pertwee, R. G. (1999). Agonist-inverse agonist characterization at CB1 and CB2 cannabinoid receptors of L759633, L759656 and AM630. *British Journal of Pharmacology*, *126*(3), 665–672. https://doi.org/10.1038/sj.bjp.0702351

Stiefl, N., Gedeck, P., Chin, D., Hunt, P., Lindvall, M., Spiegel, K., … Totrov, M. (2015). FOCUS - Development of a global communication and modeling platform for applied and computational medicinal chemists. *Journal of Chemical Information and Modeling*, *55*(4), 896–908. https://doi.org/10.1021/ci500598e

**SUPPLEMENTARY FIGURES**


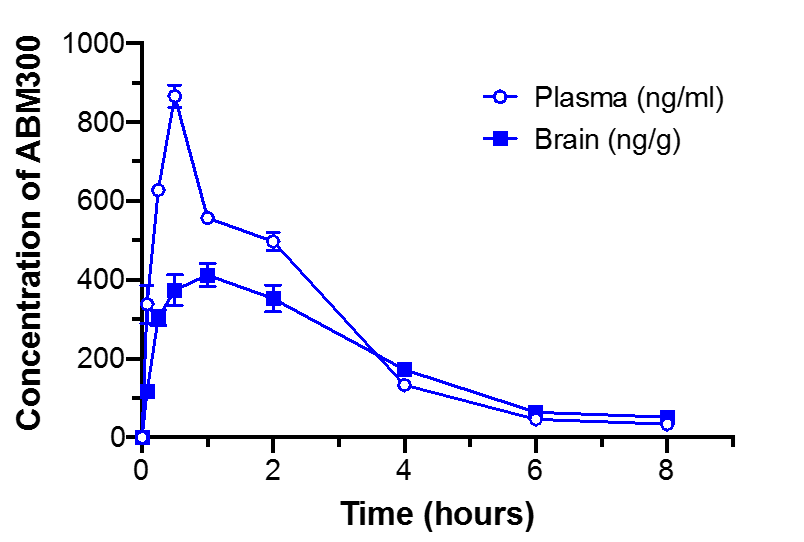


Figure S1. In vivo pharmacokinetic profile of ABM300.  Male C56BL/6 mice were administered a single intraperitoneal injection of 10 mg/kg ABM300. Samples were collected from a set of 3 mice at each of the indicated time-points, and the levels of ABM300 in plasma (ng/ml) and brain (ng/g) were quantified using LC-MS/MS as described in the Supplementary Information.  Results are shown as the mean +/- SD (n=3) of the plasma or brain concentration of ABM300.

Table S1**. In vivo pharmacokinetic profile of ABM300 following IP route of administration.**

| **Route** | **Dose**  **(mg/kg)** | **Matrix** | **T_max_**  **(hr)** | **C_max_**  **(ng/mL)** | **AUC_last_ (hr*ng/mL or hr*ng/g)** | **Ratio** |
| --- | --- | --- | --- | --- | --- | --- |
| IP | 10 | Plasma | 0.50 | 865.84 | 2054.81 |  |
|  |  | Brain^a^ | 1.00 | 411.93 | 1577.92 | 0.77 |

Table S2. **Allosteric ternary complex model of binding ([^3^H]CP 55,940) parameters at hCB_1_R CHO cell membranes.** pK_B_ is the negative logarithm of the equilibrium dissociation constant and α is the cooperativity factor of the negative allosteric modulator. Both the pK_B_ and α were determined using nonlinear regression (supplemental methods). In order to for the curve to our data the K_D_ of our radioligand was fixed to 3.5 nM and the radioligand concentration was set at 0.5. Values represent mean ± SEM of three experiments.

| **[^3^H]CP 55,940** | **Allosteric Modulator**  **ABM300** |
| --- | --- |
| pK_B_ | 6.75 ± 0.07 |
| Log α | 0.622 ± 0.08 |
| α | 4.33 ± 0.79 |

**
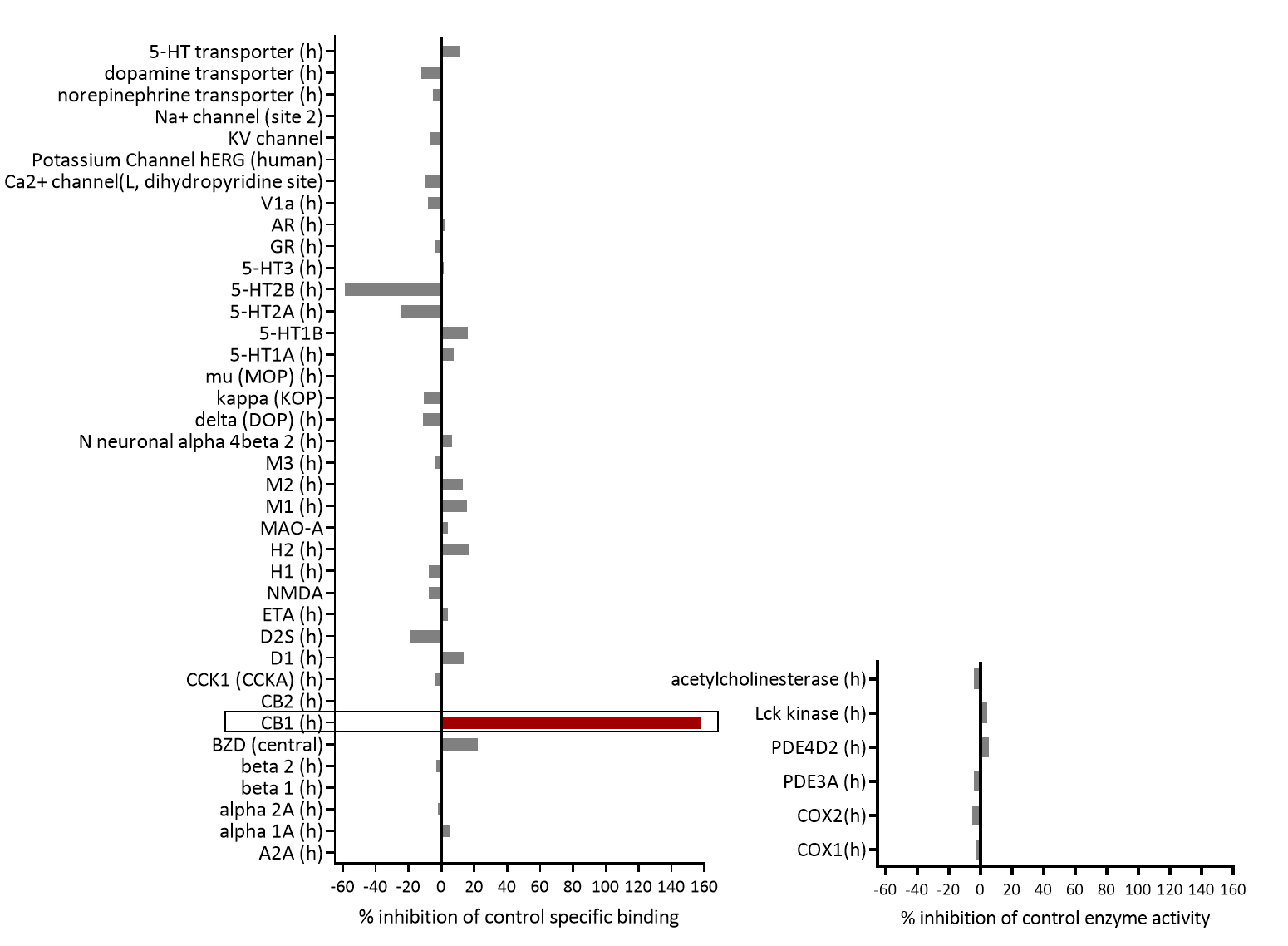
**

**Figure S2. Results of Eurofin Cerep Safety-Screen 44 Panel.** *In vitro* pharmacological profiling and assessment of the potential for off-target interactions of ABM300 in binding screens (Eurofins Cerep-Panlabs SafetyScreen 44). The compound was screened at a 1 µM concentration in duplicate for its potential to interfere with the binding of native ligands of 44 different receptors, ion channels and enzymes. For receptor assays, compound binding was calculated as percent inhibition of the binding of a radioactively labeled ligand specific for each target. For enzyme assays, the inhibition effect was calculated as percent inhibition of control enzyme activity. Tests were performed in duplicate and results are averages of those two tests.

**
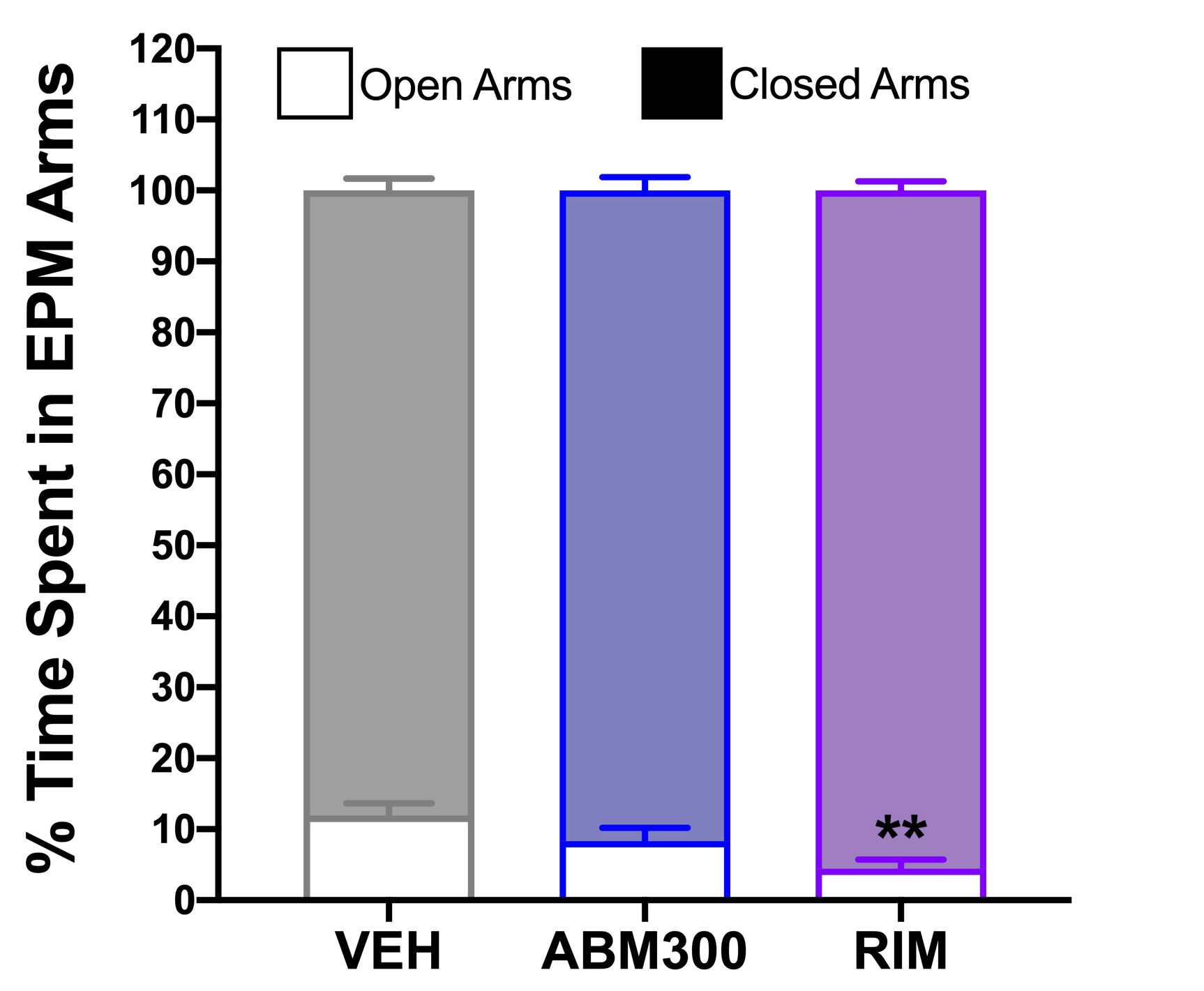
**

**Figure S3. ABM300 does not elicit anxiogenic-like side effects, as assessed in the elevated plus maze (EPM).** Rimonabant (10 mg/kg), but not ABM300 (10 mg/kg) significantly decreases the percentage of time spent in open arms in the EPM. All tests balanced for sex (n=20/drug), drugs administered 30 min before test via i.p. injection. Data shown as mean ± SEM, *****p≤0.05 compared to vehicle, **p<0.01, one-way ANOVA (open arm only), effect of treatment F[2,57]=5.268, p=0.0080, multiple comparisons, post-hoc Sidak’s test.

**
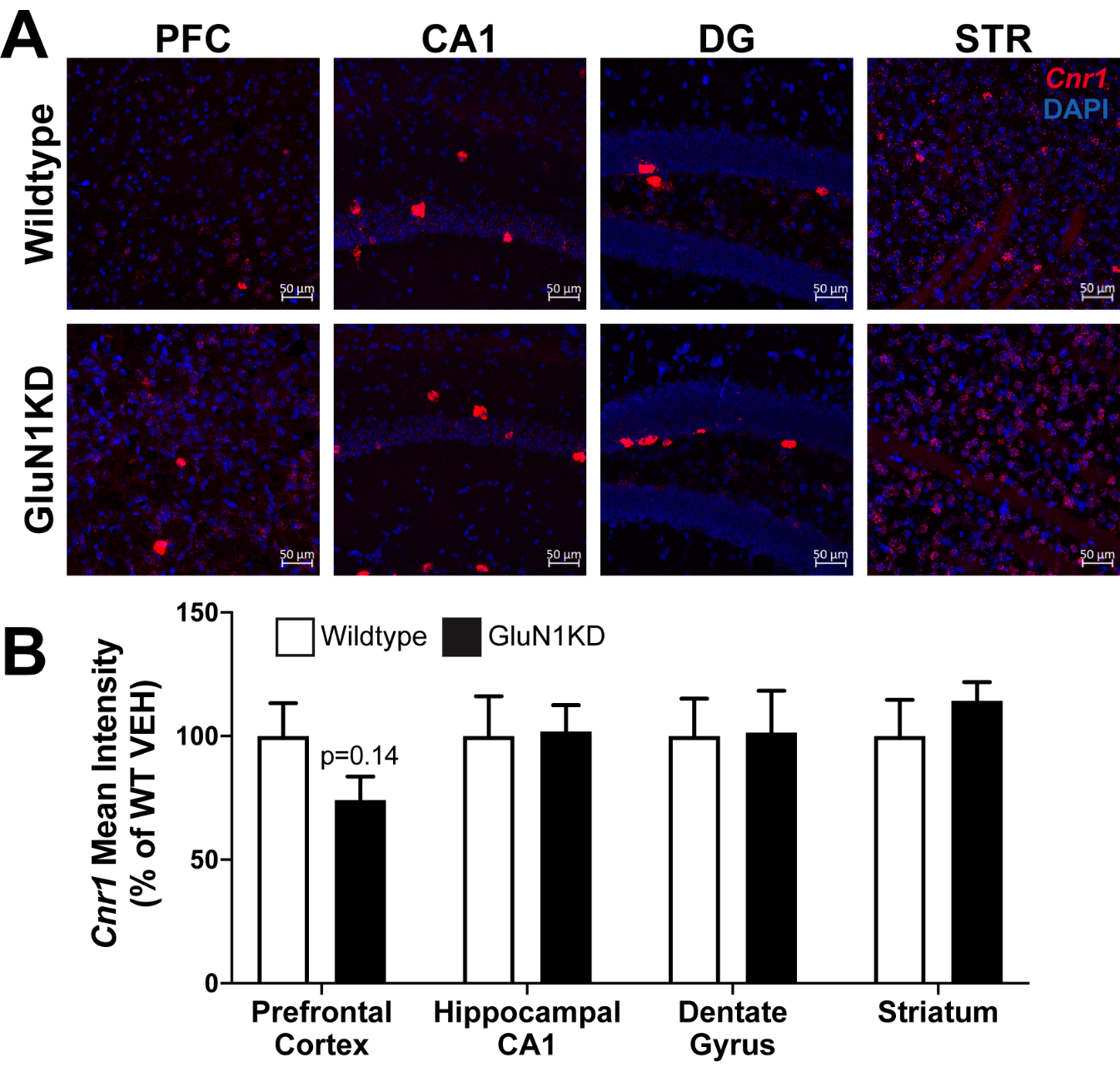
**

**Figure S4. GluN1KD mice have no change in *Cnr1* mRNA expression levels, compared to WT.** (A) Confocal images (20X) of mouse brain sagittal (20 µm) sections from WT and GluN1KD mice. Brain regions pictured: prefrontal cortex (PFC), CA1, dentate gyrus (DG) and striatum (STR). RNAscope probes visualizing mRNA of *Cnr1*, visualized in red, counter-stained with DAPI (blue). (B) Quantification of *Cnr1* mRNA expression expressed as mean pixel intensity (n=6 per genotype, balanced for sex). Data shown as mean ± SEM, Student’s t-test, within each brain region, WT vs. GluN1KD.

**
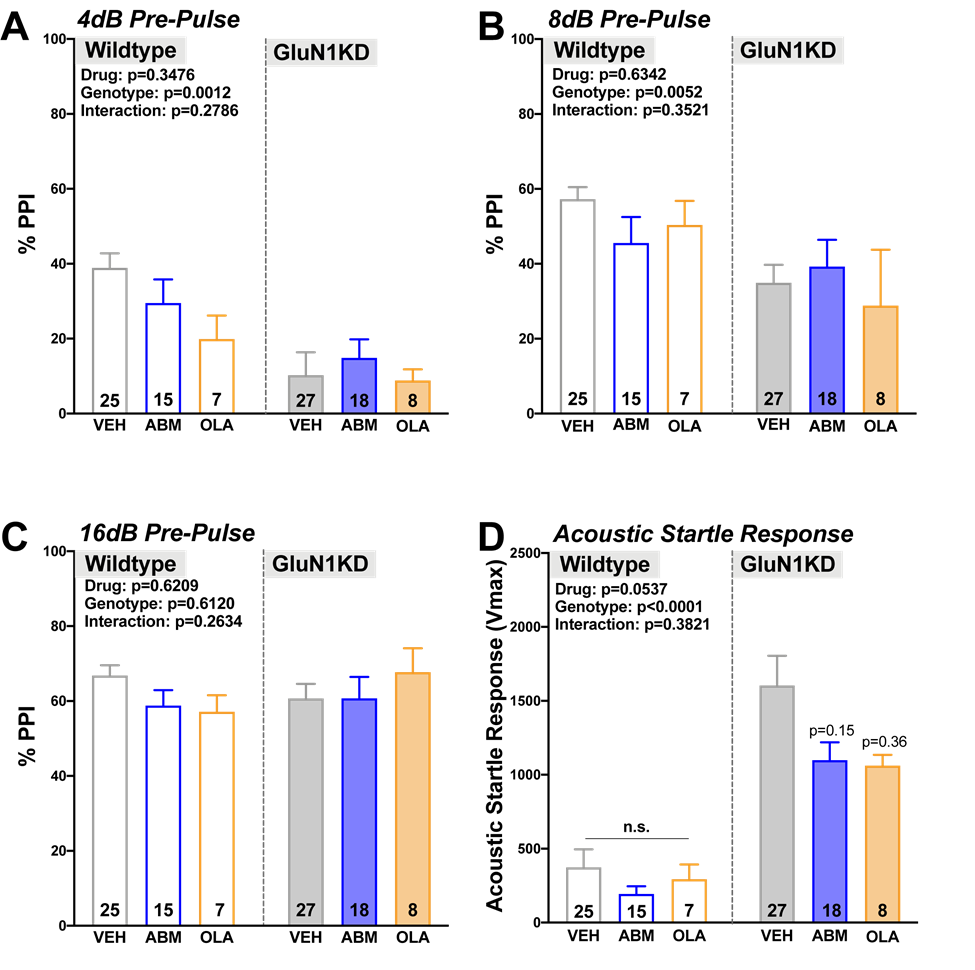
Figure S5. ABM300 has no effect on genotype-specific deficits in pre-pulse inhibition in GluN1KD mice.** There is no effect of drug (ABM300 – 10mg/kg and OLA – 1mg/kg) on genotype-specific deficits observed in sensorimotor gating (A-C) or acoustic startle response (D), tested in pre-pulse inhibition (PPI). All tests balanced for sex, drugs administered 30 min before test via i.p. injection. Data shown as mean ± SEM, *****p≤0.05 compared to vehicle (within genotype, within decibel for PPI), two-way ANOVA, multiple comparisons, post-hoc Sidak’s test. (4dB) Effect of genotype F[1,95]=11.15, p=0.0012, (8dB) effect of genotype F[1,95]=8.194, p=0.0052, and (ASR) effect of genotype F[1,95]=39.21, p<0.0001.

**
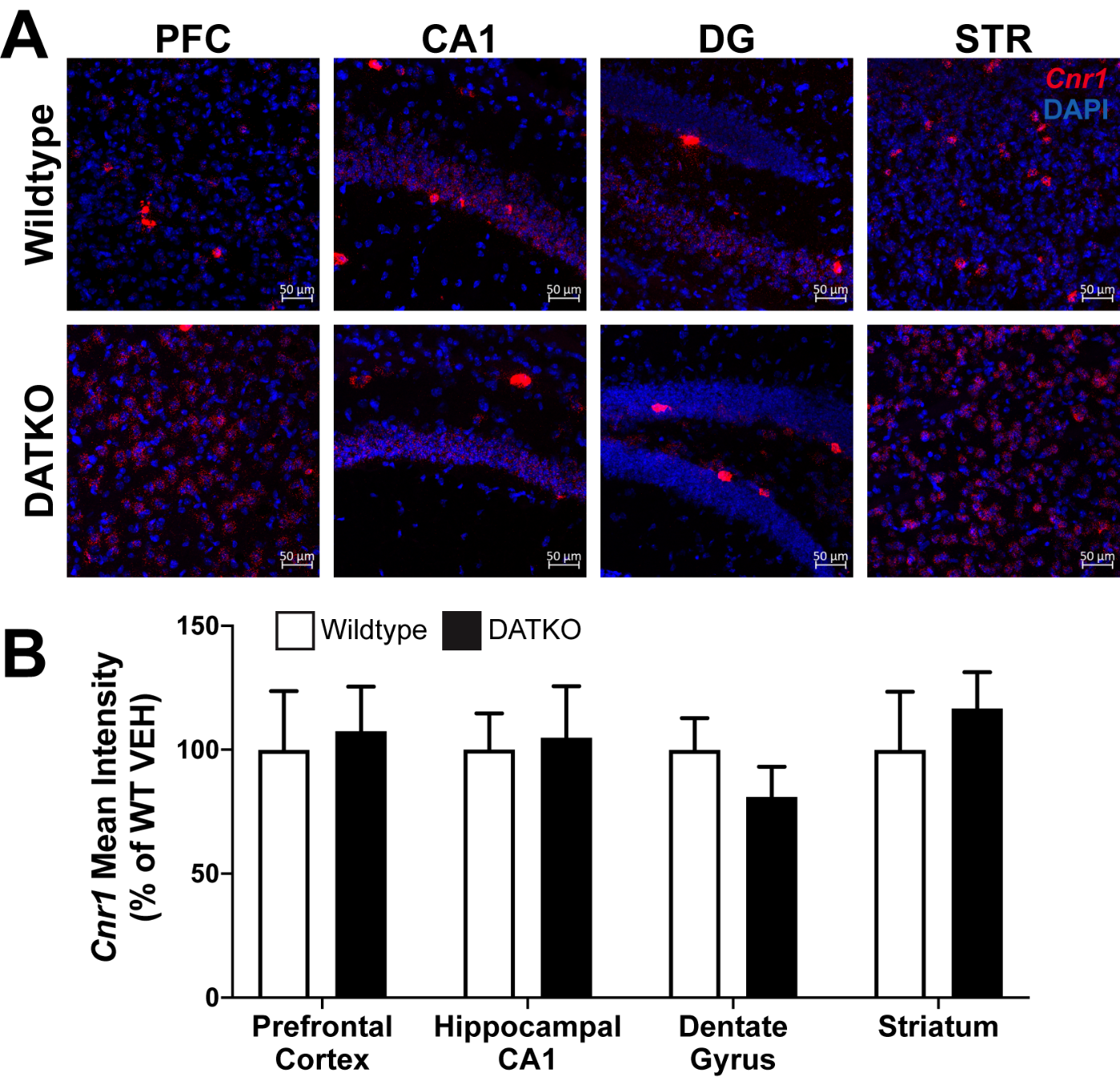
**

**Figure S6. DATKO mice have no change in *Cnr1* mRNA expression levels, compared to WT.** (A) Confocal images (20X) of mouse brain sagittal (20 µm) sections from WT and DATKO mice. Brain regions pictured: PFC, CA1, DG and STR. RNAscope probes visualizing mRNA of *Cnr1*, visualized in red, counter-stained with DAPI (blue). (B) Quantification of *Cnr1* mRNA expression expressed as mean pixel intensity (n=6 per genotype, balanced for sex). Data shown as mean ± SEM, Student’s t-test, within each brain region, WT vs. DATKO.

**
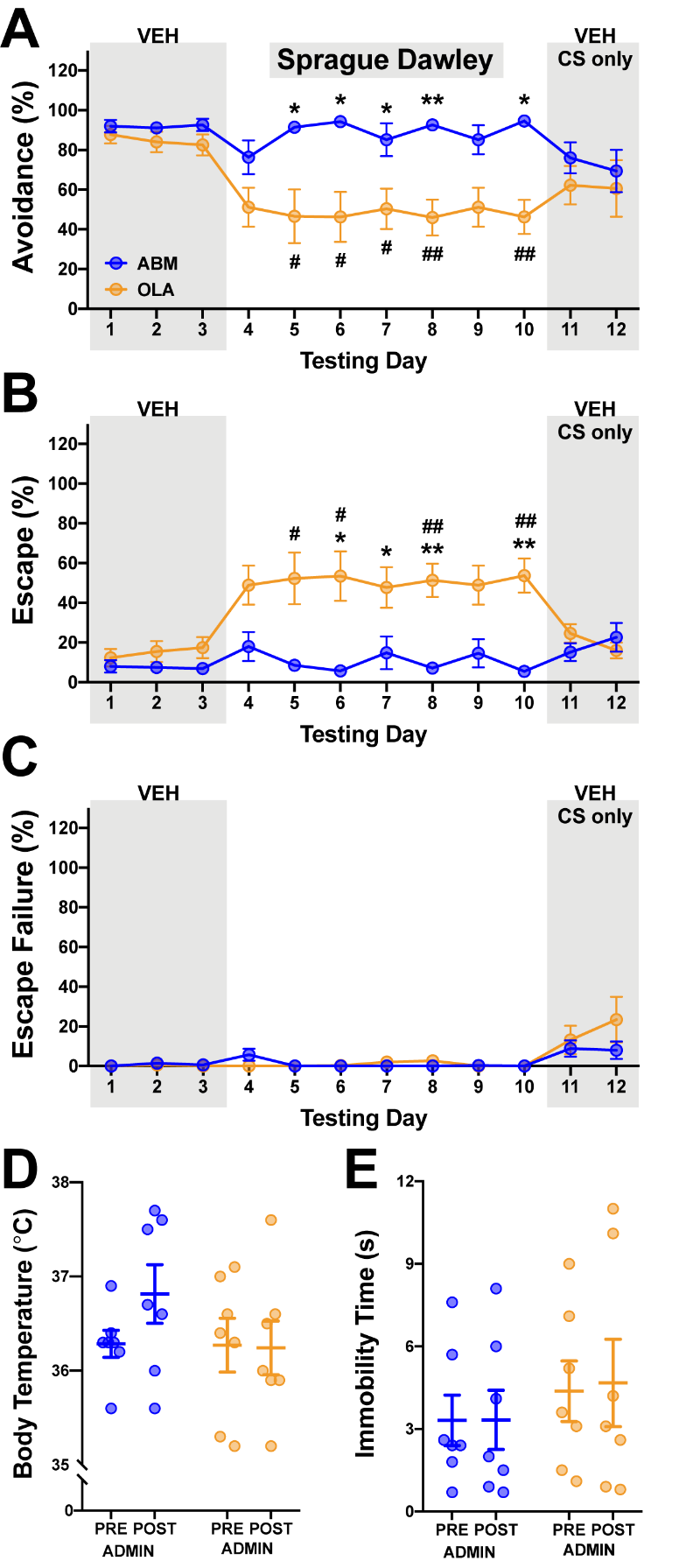
Figure S7. Treatment with olanzapine (1mg/kg), but not ABM300 (10mg/kg), attenuates avoidance behaviour and enhances escape responding in CAR testing.** Effect of ABM300 (ABM; n=7) and olanzapine (OLA; n=7) on shock avoidance (A), shock escape (B), and escape failures (C) in CAR. Baseline response following vehicle injection (Days 1-3) and response after drug injection (Day 4-10). Re-emergence of avoidance behaviour under CS-only conditions evaluated following drug administration regimen (Days 11-12). Data shown as mean ± SEM, **^#^**p≤0.05 group difference between baseline Day 3 and drug testing data (^#^p<0.05, ^##^p<0.01), *****p≤0.05 vehicle baseline data used as covariates to determine main effects, interactions, and between group differences (*p<0.05, **p<0.01), RM ANOVA with Bonferroni correction.
